# Modelling Holocene analogues of coastal plain estuaries reveals the magnitude of sea-level threat

**DOI:** 10.1038/s41598-019-39516-4

**Published:** 2019-02-25

**Authors:** Anna M. Helfensdorfer, Hannah E. Power, Thomas C. T. Hubble

**Affiliations:** 1 School of Geosciences, The University of Sydney, Sydney, NSW 2006 Australia; 2 School of Environmental and Life Sciences, The University of Newcastle, Callaghan, NSW 2308 Australia

## Abstract

Hydrodynamic modelling of Australia’s lower Murray River demonstrates the response of a large coastal plain estuary to the mid-Holocene (7,000–6,000 yr BP) sea-level highstand. The approximately two metre higher-than-present sea level during the highstand forced the estuarine limit upstream generating an extensive central basin environment extending more than 200 kilometres from the river mouth (143 kilometres upstream of the modern tidal limit). The geomorphic history of the region does not conform to conventional estuarine facies models as, for much of the Holocene, the lower Murray River acted as a landward, gorge-confined extension of the Murray estuary. The incredibly low relief of this coastal plain system drove significant saline incursion and limited current velocities across the estuary facilitating deposition of a laminated silt-clay sequence which our results suggest may be regionally extensive. Variations to discharge, barrier morphology, or the estuary’s bathymetry result in minimal change to the estuarine palaeo-environment. The shift to the present-day fresher water distribution in the Murray estuary requires a fall in sea level to present-day conditions. The dominance of sea level as the controlling factor on this estuarine palaeo-environment highlights the significant potential impact of climate change induced sea-level rise to coastal plain estuaries.

## Introduction

Coastal plains and lowlands are characterised by their low gradient and commonly dense populations, with geomorphic-based risk assessments revealing their significant vulnerability to future climatic change^1^. Inundation associated with an increase in mean sea level threatens communities, coastal infrastructure and ecosystems, with estuaries vulnerable to the compounding influences of storm surges and strong winds, along with implications of saline incursion for irrigation and drinking water supply^2^. Indeed, in Australia, flooding is considered the most significant medium-term climate change hazard, with a shift to coastal inundation beyond mid-century^2,3^. There is, however, less emphasis on the consequences of rising sea levels for saline intrusion, particularly for low-gradient coastal plain estuaries.

The Intergovernmental Panel on Climate Change (IPCC) projects that global mean sea level will rise by 0.53–0.97 m by 2100 under a high emissions scenario, with these projections likely to be exceeded by at least 10% in Australia^2^. Crucially, even given a stabilisation in temperatures, global mean sea level will continue to rise for several centuries beyond 2100^2^. Understanding the dominant drivers of environmental change within an estuarine system allows for effective management given uncertainties in future mean sea level, and determining palaeo-environmental responses to the Holocene highstand provides a useful analogue of expected change. There is a pressing need for palaeo-environmental analysis in economically significant regions to direct future natural resource management policies, particularly in intensively managed environmental systems. Developing appropriate management strategies that negate the detrimental impacts forecast in climate change projections is particularly important for lowland coastal plains where rising sea levels will undoubtedly cause problematic inundation and saline intrusion. Applying Holocene analogues to future sea-level rise scenarios is a well-recognised approach to predicting responses of coastal systems to climate change^4,5^. Here, we use the lower Murray River (LMR) and Murray estuary as a case study to demonstrate the utility of understanding Holocene analogues to plan for potential environmental change in coastal plain estuaries.

**Figure 1:**
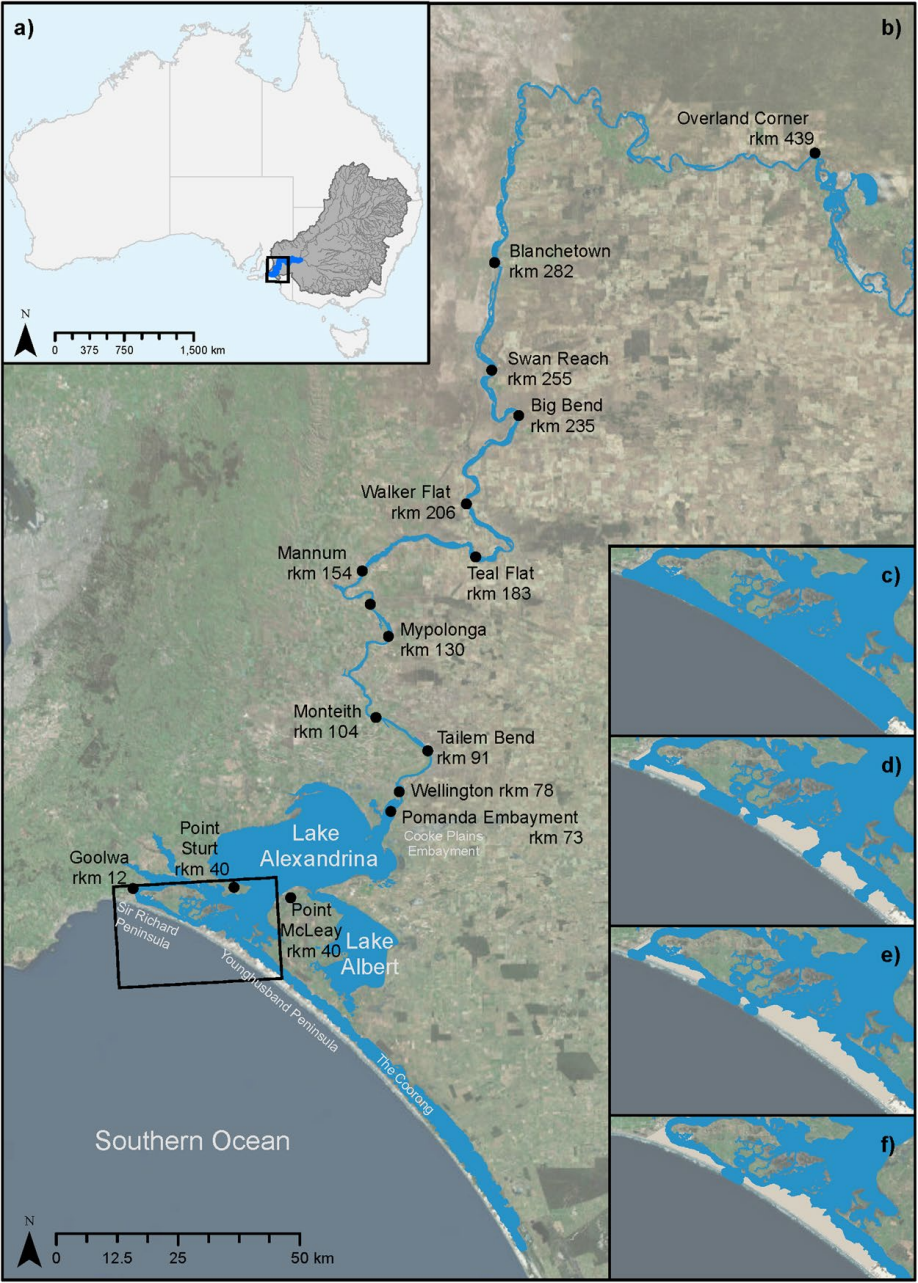
Study area and modelled barrier morphologies. (**a**) The Murray Darling Basin (grey) is Australia’s largest and most politically and economically important river basin. The Murray Darling Basin comprises the Darling and Murray River catchments, whose major watercourses are shown in dark grey. The lower Murray River (blue) is the final segment of this system flowing from the confluence of the Darling and Murray Rivers at Wentworth, New South Wales, to the Murray Mouth at Goolwa, South Australia. (**b**) Within South Australia, the lower Murray River flows through the confines of the Murray Gorge from Overland Corner to Wellington before debouching into Lake Alexandrina at the Pomanda Embayment. The lower Murray River reaches the Southern Ocean at the Murray Mouth between Sir Richard and Younghusband Peninsulas. Collectively, Lakes Alexandrina and Albert are known as the Lower Lakes, and together with the Coorong and Murray Mouth, form the modern-day Murray estuary. The four modelled barrier morphologies, accounting for the chain-of-islands Holocene evolution of Sir Richard and Younghusband Peninsulas^13,31,37,46^, are depicted by outlets to the ocean (blue) and barrier features (brown) in (**c**) B_0_; (**d**) B_+_ and (**e**) B_++_, with (**f**) B_mod_ representing the modern-day Murray Mouth. Satellite imagery source: ESRI.

Understanding fluvial and estuarine responses to sea-level cycles through their associated depositional systems tracts may assist in predicting potential impacts of future sea-level rise. Research has shown that fluvial systems attempt to keep pace with changing base level, with shoreline advance or retreat controlling available accommodation and causing a shift in the nature and location of estuarine processes and depositional environments^5^. There is a significant body of literature detailing the influence of Holocene sea-level change on the sedimentary infill and evolution of estuaries on the east coast of Australia^6–11^, however, few studies specifically examine southern coast Australian estuaries, such as the Murray estuary (e.g^12–14^.). The Murray estuary lies at the terminus of the Murray-Darling Basin (MDB), Australia’s largest and most politically and economically important river basin (Fig. 1). The geomorphic and palaeo-environmental history of the region has been the subject of much debate, driven by the reliance on Holocene climatic and hydrologic reconstructions to guide nationally significant water policy^15^. This wave-dominated estuary, comprising the Lower Lakes, Coorong and Murray Mouth, is situated on a low-gradient coastal plain and developed in response to slowing sea-level rise during the transgressive period of the early- to mid-Holocene^16–18^. The Murray estuary’s barrier complex, comprising Sir Richard and Younghusband Peninsulas, began formation at approximately 8,000 yr BP, allowing for the development of the central basin lakes, Alexandrina and Albert, prior to the Holocene highstand at 7,000–6,000 yr BP^16–21^ (Fig. 1).

Upstream, the LMR is entrenched within the Murray Gorge (from Overland Corner to Wellington, Fig. 1), with the valley fill only comprising sediment of the most recent cycle of lowstand, transgression and highstand^5,22^. This consists of two distinct facies: (1) the Monoman Formation’s coarse-grained sands comprise the lower valley fill, and (2) the Coonambidgal Formation’s fine-grained clays and silts comprising the upper valley fill. The Holocene infill of Lake Alexandrina is known as the St Kilda Formation, and is a finely laminated silt-clay sequence^23,24^. Deposition of this sequence had commenced by at least 8,000 yr BP^23,24^; however, it is probable that deposition within the palaeo-channel that transited through modern-day Lake Alexandrina had commenced prior to 8,000 yr BP as dated cores within this area bottom out on laminated mud^23,24^. The St Kilda Formation was regionally extensive from 5,500 yr BP and has characterised the sediments of Lake Alexandrina since^23,24^.

Standing water or very weak current velocities are required for the deposition and preservation of a laminated silt-clay sequence^25^. However, recent flume studies have demonstrated mud floc deposition as distinct laminae in current velocities up to 0.3 m/s, with laminae accumulation considered possible at higher velocities given particularly high sediment concentrations^26,27^. Laminations, such as those present throughout Lake Alexandrina, are undoubtedly a product of low energies and high sedimentation rates^28^, features characteristic of central basin environments^29^. Indeed, wave-dominated estuaries are known for their well-defined tripartite zonation of facies assemblages with lithofacies typically presenting a coarse-fine-coarse sequence: the marine sands of the barrier complex and flood-tide delta, the clays and silts of the central basin, and the fluvial sands of the bayhead delta and river channel^30^. Conventional presentations of estuarine models indicate that the point where the river debouches into the lagoon locates the transition to reduced energy and defines the landward extent of the central basin and seaward extent of the bayhead delta. However, the contentious Holocene palaeo-salinities of the Lower Lakes give rise to debate on the extent and character of the Murray estuary. Some place the upstream extent of this estuary at the Pomanda Embayment, where the LMR debouches into Lake Alexandrina^16^ (river kilometre (rkm) 73, Fig. 1), with other authors even suggesting that the Lower Lakes were freshwater stilling basins for the duration of the Holocene and cannot be classified as part of this estuarine zone^17^.

In this paper, we evaluate the range of possible responses of the palaeo-Murray estuary to the Holocene sea-level highstand. Specifically, we:

- Conduct hydrodynamic modelling of the palaeo-Murray estuary and LMR with sensitivity testing for discharge, bathymetric surface and barrier morphology with results analysed for inundation extents, water levels and depths, flow velocity and salinity.
- Assess the palaeo-environment that likely prevailed during the Holocene highstand and correlate model scenarios with geomorphic and sedimentary features of the region to develop a model of estuarine processes zonation and inferred resulting morphology.
- Assess the relative influence of geomorphic and hydrodynamic drivers on the estuary during the Holocene sea-level highstand.
- Propose the palaeo-Murray estuary, as a possible end-member exemplar of an extremely low-gradient coastal plain estuary, to demonstrate the significant threat of sea-level rise due to climate change on the environmental character of coastal plain estuaries. Particular reference is given to the understated potential impact of climate change-induced saline intrusion on the character and extent of coastal plain estuaries.

## Results

Here, we model the inundation extents, water depths, flow velocities and salinities for the full extent of the LMR and Murray estuary as a function of four key morphologic and hydrodynamic forcings: (1) bathymetric surface (two end members and a best estimate), (2) sea level (Holocene highstand and present-day), (3) discharge (drought, pre-regulation average, and flood), and (4) barrier morphology (four scenarios, ranging from completely open to almost closed, to account for barrier evolution). Results are grouped into six categories based on bathymetric surface and sea level: S_low_WL_2_, S_low_WL_0_, S_mid_WL_2_, S_mid_WL_0_, S_up_WL_2_ and S_up_WL_0_ (see *Methodology - Overview of model result categories* and Table S1 for further information).

### Model correlation with regional geomorphology and sedimentology

**Figure 2:**
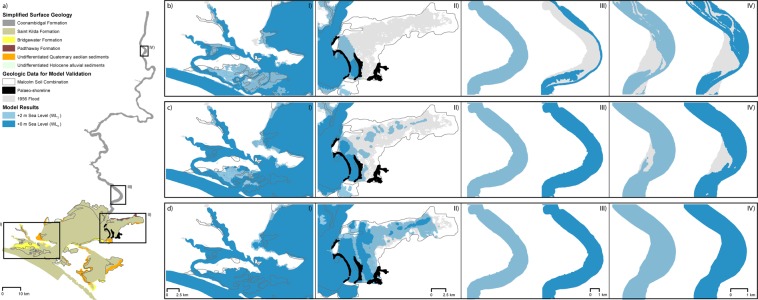
Geologic overview of the study area and maps showing maximum inundation extent under average discharge and modern-day barrier conditions (D_av_B_mod_ scenarios). (**a**) Simplified surface geology showing Holocene and Pleistocene stratigraphic formations. Inundation extents are shown for (**b**) S_up_, (**c**) S_mid_ and (**d**) S_low_ scenarios at +2 m sea level (WL_2_; light blue) and +0 m sea level (WL_0_; dark blue). Panels I and II encompass sub-sections of Lake Alexandrina detailing the flood tide delta and Murray Mouth, and the Cooke Plains Embayment respectively. Panel III details a 10 rkm representative sub-region of the lower portion of the LMR (centered on Tailem Bend, rkm 91). Panel IV details a 10 rkm representative sub-region of the upper portion of the LMR (centered on Swan Reach, rkm 255). The inundation extent of the 1956 flood (grey) is given as an indicative regional modern-day analogue of the plausible extent of inundation caused by backfilling during the Holocene highstand. The Malcolm soil combination (dark grey outline) represents the maximum Holocene inundation extent of the Lower Lakes (Lakes Alexandrina and Albert; I and II)^31^. Palaeo-shorelines (black) allude to the maximum Holocene extent of Lake Alexandrina and a period of stability following retreat to the present-day shoreline (II)^31^. Maximum inundation extents remain comparable in other model scenarios not depicted here, with non-significant fluctuations in inundation across the Lower Lakes (I and II), with the exception of a significant flood event (D_+_ scenarios) which induces valley-wide inundation throughout the LMR (III and IV), consistent with 1956 flood extents.

Given the experimental nature of modelling snapshots in geological time, constraining inundation extent to geological features gives an indication of the plausibility of model results. The lacustrine and estuarine clays of the Malcolm soil combination (Fig. 2a; Table S2) represent the extent of Lake Alexandrina during the Holocene^31^. This formation, and recognised Holocene palaeo-shorelines^31^, are mapped against inundation extent in Fig. 2. The Malcolm soil combination is well constrained by the S_low_WL_2_ scenarios (Fig. 2d; Table S1), with the S_mid_WL_2_ scenarios proving a reasonable match overall (Fig. 2c; Table S1). The S_low_WL_2_ scenarios align precisely with the most expansive of the palaeo-shorelines which is consistent with the greatest Holocene extent of Lake Alexandrina^31^ (Fig. 2d; Table S1). The S_mid_ scenarios sit at, or beyond, the middle palaeo-shoreline, considered to represent a short stabilisation period during retreat, likely in response to falling sea levels^31^ (Fig. 2c; Table S1). The S_up_WL_2_ scenarios align with the middle palaeo-shoreline, while the S_up_WL_0_ scenarios generate inundation akin to present-day^31^ (Fig. 2b; Table S1).

The inundation extent of the 1956 flood, the greatest flood on instrumental record, is also depicted in Fig. 2. This extent is not as expansive as the Malcolm soil combination within the Lower Lakes region, suggesting that water levels at the Holocene highstand were well above maximum historical records. Bank overtopping from fluvial floodwaters during the 1956 flood caused valley-wide inundation within the Murray Gorge which gives insight into the plausible response to the Holocene highstand as the LMR is backfilled. All S_low_ scenarios correlate with the 1956 flood extent and are characterised by valley-wide inundation throughout the Murray Gorge (Fig. 2d III-IV; Table S1). The S_mid_WL_2_ scenarios inundate the entire valley, with the exception of two small areas at Big Bend (rkm235) and Swan Reach (rkm255; Fig. 2c III-IV; Table S1). Inundation of these two locations is reduced in the S_mid_WL_0_ scenarios along with isolated small dry areas, however, these models remain characterised by valley-wide inundation (Fig. 2c III-IV; Table S1). Conversely, even given Holocene highstand sea levels, the S_up_ scenarios are charactered by a channel with fringing swamps upstream of Mypolonga (rkm 130), as was evident prior to levee construction and land reclamation in the 19^th^ century^32^. A significant flood (D_+_ scenarios) is required to induce valley-wide inundation (Fig. 2b III-IV; Table S1). Our results suggest that the period of sea-level fall from highstand in the late-Holocene saw a significant shift in the geomorphic character of the LMR (Fig. 2c III-IV vs. 2b III-IV). Overall, model results are well correlated to regional geomorphology and sedimentology, and are consistent with research into the sedimentary infill and geomorphological evolution of barrier estuaries identified on the east coast of Australia (e.g^7,8,33^.), therefore, the model is deemed sensible and valid as a basis for further exploratory analysis.

### Palaeo-environment at the Holocene highstand

**Figure 3:**
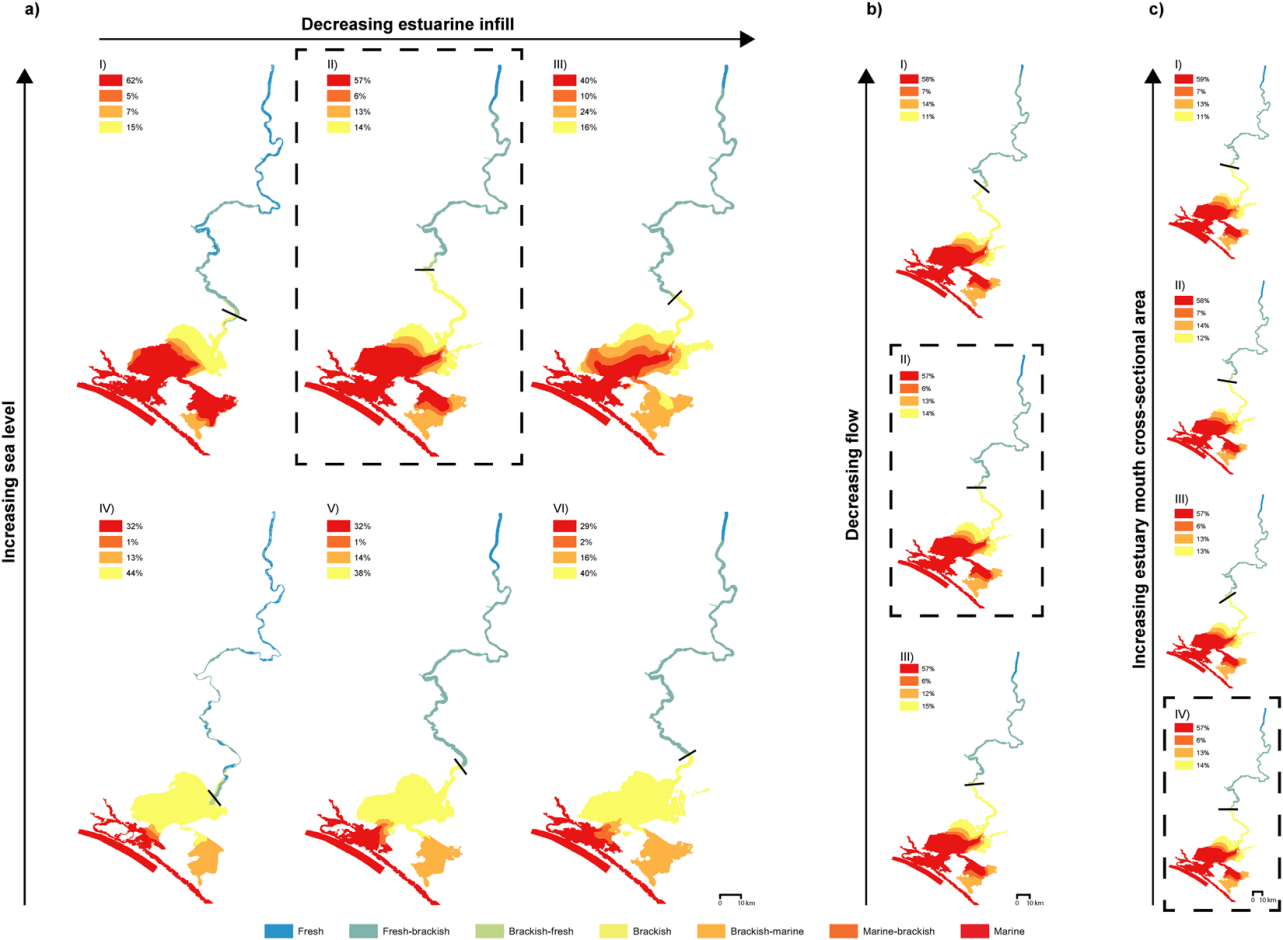
Key representative maps comparing maximum salinity reached relative to sea level, bathymetric surface, discharge and barrier morphology. (**a**) An increase in sea level from WL_0_ present-day conditions (IV–VI) to WL_2_ Holocene highstand conditions (I–III) significantly increases marine incursion, extending to the upper reaches of Lake Alexandrina and pushing the brackish limit further up the Murray Gorge. There is negligible change to the overall palaeo-environmental character of the region between end-member and best-estimate Holocene bathymetries (I–III or IV–VI). (**b**) Variance in flow from drought (D_−_) to flood (D_+_) scenarios (I–III) is unable to alter the palaeo-environmental character of the region. (**c**) Variance in barrier morphology from completely open (B_0_) to modern-day (B_mod_) outlet scenarios (I–IV) is also unable to alter the palaeo-environmental character of the region. The isohaline (black line) delimits the brackish limit (equivalent to 1 psu) with the percentage area of each salinity class seaward of the isohaline given relative to total inundated area. The hatched box highlights the common scenario between the three panels: scenario S_mid_WL_2_D_av_B_mod_. Within (**a**) all maps shown are pre-regulation average discharge with modern-day barrier morphology scenarios (I: scenario S_up_WL_2_D_av_B_mod_; II: scenario S_mid_WL_2_D_av_B_mod_; III: scenario S_low_WL_2_D_av_B_mod_; IV: scenario S_up_WL_0_D_av_B_mod_; V: scenario S_mid_WL_0_D_av_B_mod_ and VI: scenario S_low_WL_0_D_av_B_mod_; Table S1). To demonstrate representative salinities at the Holocene highstand, S_mid_WL_2_ scenarios are shown within (**b**) (I: scenario S_mid_WL_2_D_-_B_mod_; II: scenario S_mid_WL_2_D_av_B_mod_; III: scenario S_mid_WL_2_D_+_B_mod_) and (**c**) (I: scenario S_mid_WL_2_D_av_B_0_; II: scenario S_mid_WL_2_D_av_B_+_; III: scenario S_mid_WL_2_D_av_B_++_; IV: scenario S_mid_WL_2_D_av_B_mod_). Salinity is measured based on the classification scheme of Tooley^51^.

**Figure 4:**
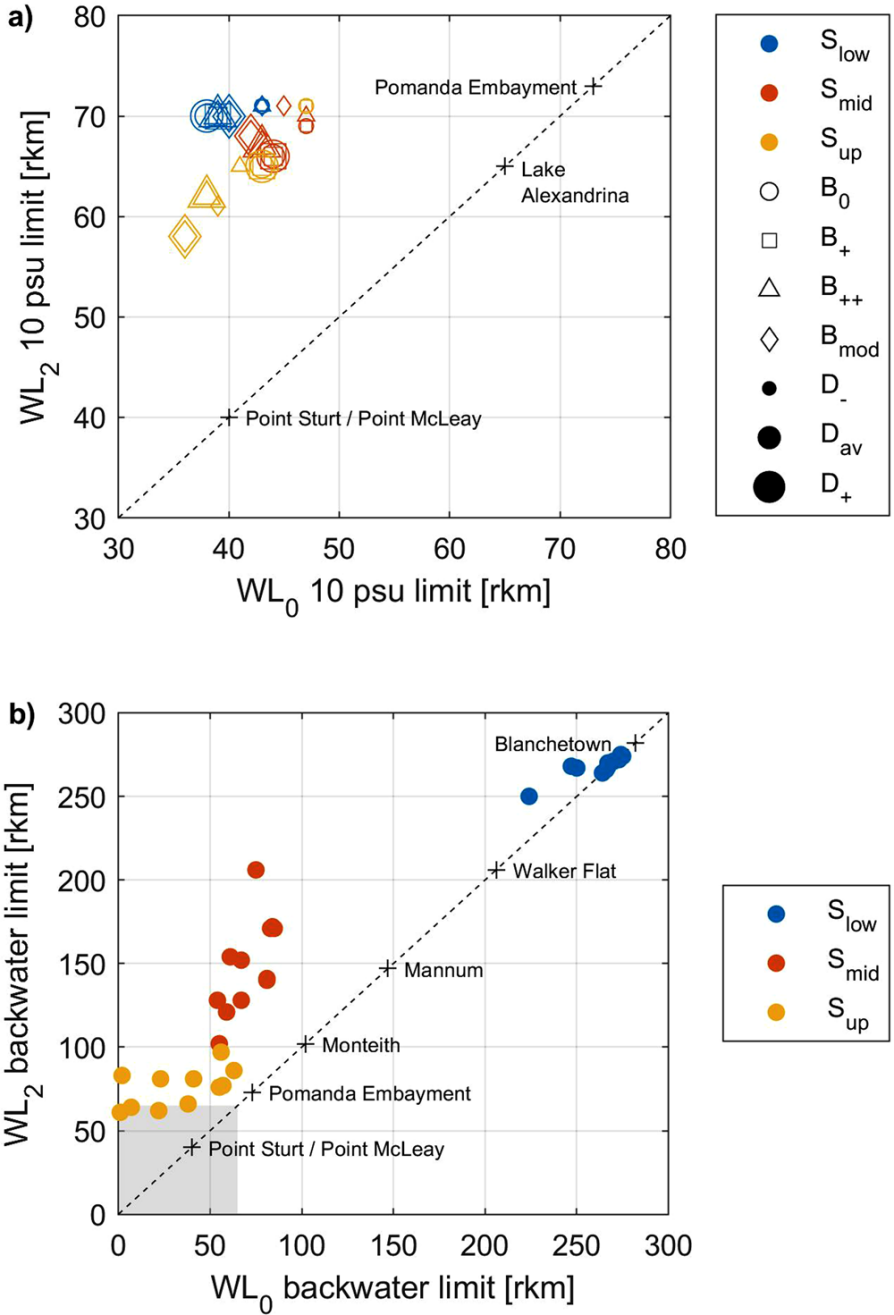
Comparison of 10 psu (marine-brackish) and backwater zone limits for all WL_0_ and WL_2_ paired scenarios. (**a**) An increase in sea level from WL_0_ to WL_2_ Holocene highstand conditions drives the 10 psu (marine-brackish) limit from the flood tide delta and lower reaches of Lake Alexandrina upstream such that marine or marine-brackish waters occupy the entire body of the lake. This trend is apparent irrespective of bathymetric surface (marker colours), barrier morphology (marker shapes) or discharge (marker sizes) demonstrating that sea level is the primary driver of palaeo-environmental change within this system. (**b**) Velocity vector convergence, taken as the point of convergence of upstream and downstream velocity vectors within the channel thalweg, defines the upstream extent of the backwater zone. Given S_up_ conditions (yellow), the backwater zone is restricted to the main body of Lake Alexandrina (grey shading) and the Pomanda Embayment within WL_0_ scenarios, with the higher sea level in WL_2_ scenarios driving this limit upstream into the lower reaches of the Murray Gorge. The influence of sea level on the backwater limit is equally apparent given S_mid_ conditions (red), where at the Holocene highstand, an enlarged low energy backwater setting was emplaced up to Walker Flat (rkm 206). By comparison, the S_low_ surface (blue) forces the backwater zone to occupy nearly the entire model domain irrespective of sea level.

Our results show that the palaeo-environment at the Holocene highstand was likely to have been estuarine throughout the Lower Lakes and well upstream into the LMR (Figs 3a I–III, 4a and S1a–c). All WL_2_ highstand scenarios result in an estuarine environment with significant marine incursion in the Lower Lakes, meanwhile all WL_0_ scenarios result in a brackish environment within the Lower Lakes, with fluvial discharge supressing significant marine incursion to the barrier and flood tide delta complex (Figs 3a, 4a and S1). This trend is apparent across all scenarios irrespective of discharge, barrier morphology and bathymetric surface (Figs 3, 4a and S1), with a shift to fresher water dependent upon a fall in sea level to present-day conditions (Figs 3a, 4a and S1). Holocene highstand sea levels also induce valley-wide inundation under S_up_ morphology, with the S_mid_ and S_low_ morphologies resulting in a significant increase in the areal extent of the Lower Lakes (Fig. 2). These results demonstrate that sea level is the driving factor controlling the environmental character of the Lower Lakes and LMR. This is apparent through the difference in maximum palaeo-salinities observed with a change in sea level (Figs 3a, 4a and S1) when compared with the near-identical results produced by sensitivity testing for discharge (Figs 3b and 4a), barrier morphology (Figs 3c and 4a) or bathymetric surface (Figs 3a and 4a).

### Estuarine processes zonation and inferred resulting morphology

Flow velocity vectors are used to define the upstream extent of the backwater zone for each scenario (Figs 4b and S2). The maximum upstream extent in S_low_ scenarios is Blanchetown (rkm 275), regardless of sea level, such that the S_low_WL_0_ scenarios present significantly different backwater zones when compared to other WL_0_ scenarios (Figs 4b and S2; Table S1). Given that the bathymetry of the S_low_ scenarios is almost certainly not representative of the mid- to late-Holocene when sea levels had receded to present-day, the backwater zone during this period is best constrained by the S_mid_WL_0_ and S_up_WL_0_ scenarios, confining the backwater zone to the region downstream of Tailem Bend (rkm 91; Figs 4b and S2; Table S1). Under all bathymetric conditions, the backwater zone extended well into the LMR supporting the hypothesis that Lake Alexandrina and the LMR were subject to a single depositional environment that produced a regionally extensive central basin depositional sequence at the Holocene highstand (Figs 4b and S2). We suggest that, prior to anthropogenic modifications of the flow regime, this central basin sequence was continuing to accumulate within the entirety of Lake Alexandrina; top-of-core modern dates across the regionally extensive laminated sequence support this hypothesis^23^.

**Figure 5:**
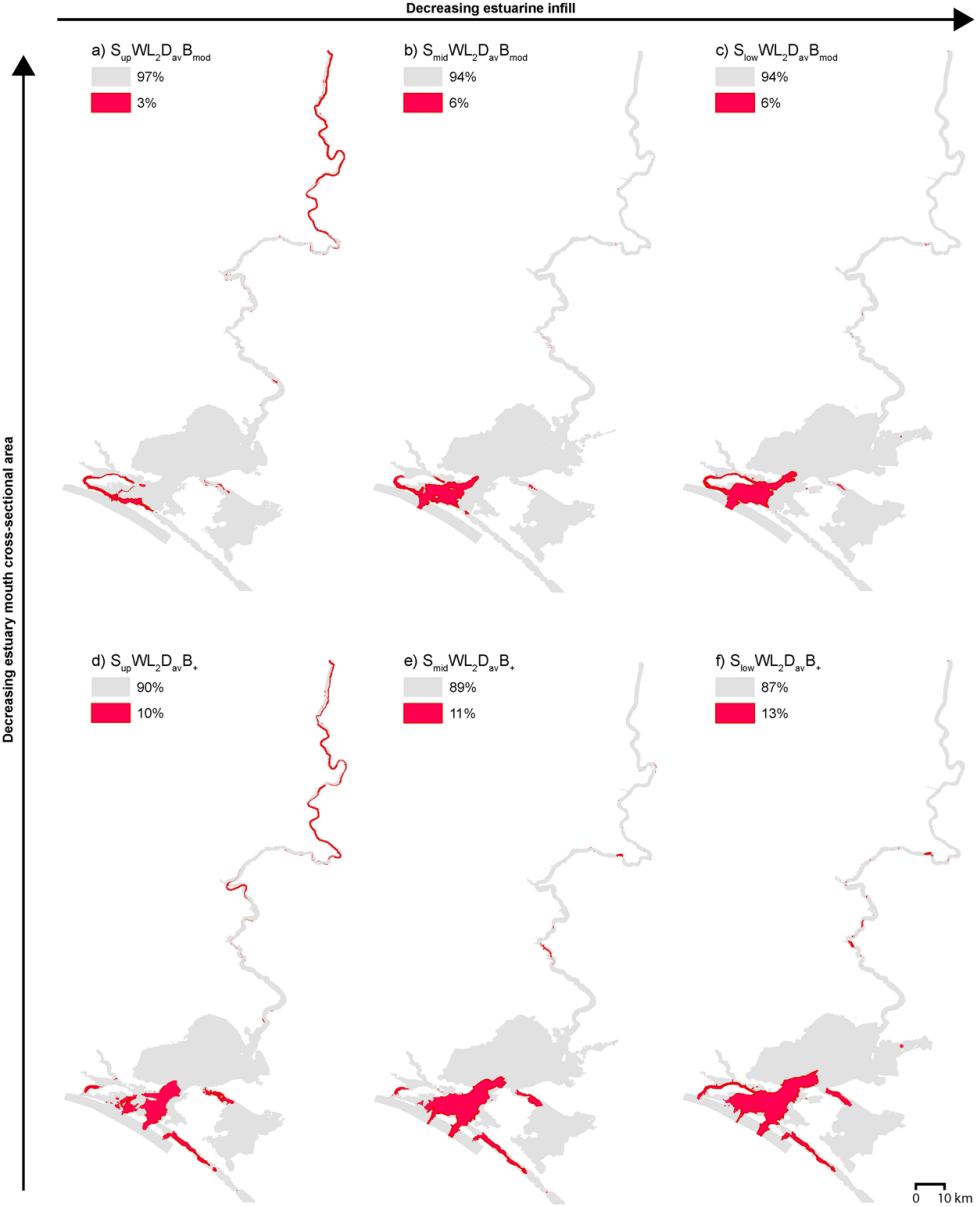
Key representative maps comparing maximum flow velocity magnitude relative to barrier morphology and bathymetric surface. Areas are shaded red where maximum velocity >0.3 m/s and conditions are therefore not conducive to the deposition of a laminated silt-clay sequence^25–27^. Barrier evolution from B_mod_ to B_+_ (a–c to d–f respectively) has a minimal influence on the area conductive to velocities <0.3 m/s, while variance in bathymetry from S_low_ (**c** and **f**), or S_mid_ (**b** and **e**) to S_up_ (**a** and **d**) increases velocities within the back swamps upstream of Teal Flat (rkm 183), with comparable velocities elsewhere in the model domain. All scenarios shown are WL_2_ (+2 m sea level) scenarios, demonstrating representative velocities at the Holocene highstand: (**a**) scenario S_up_WL_2_D_av_B_mod_; (**b**) scenario S_mid_WL_2_D_av_B_mod_; (**c**) scenario S_low_WL_2_D_av_B_mod_; (**d**) scenario S_up_WL_2_D_av_B_+_; (**e**) scenario S_mid_WL_2_D_av_B_+_; (**f**) scenario S_low_WL_2_D_av_B_+_.

The possibility of deposition and preservation of a laminated sequence is limited to regions where the maximum flow velocity magnitude is <0.3 m/s^25–27^ (see grey shaded areas in Figs 5 and S3), which encompasses a minimum of 82% of the model domain. Suitable conditions for the deposition of a laminated sequence throughout Lake Alexandrina apply in all scenarios (Figs 5 and S23) and explain the regionally extensive presence of this laminated central basin deposit that has characterised Lake Alexandrina’s Holocene stratigraphy since 5,500 yr BP^23^. Variation in barrier morphology (Fig. 5a–c or d–f) or LMR/Lower Lakes bathymetry (Fig. 5a,d or b,e or c,f) makes a negligible difference in the regional capacity to generate a laminated central basin deposit (maximum 7% and 3% change respectively).

**Figure 6:**
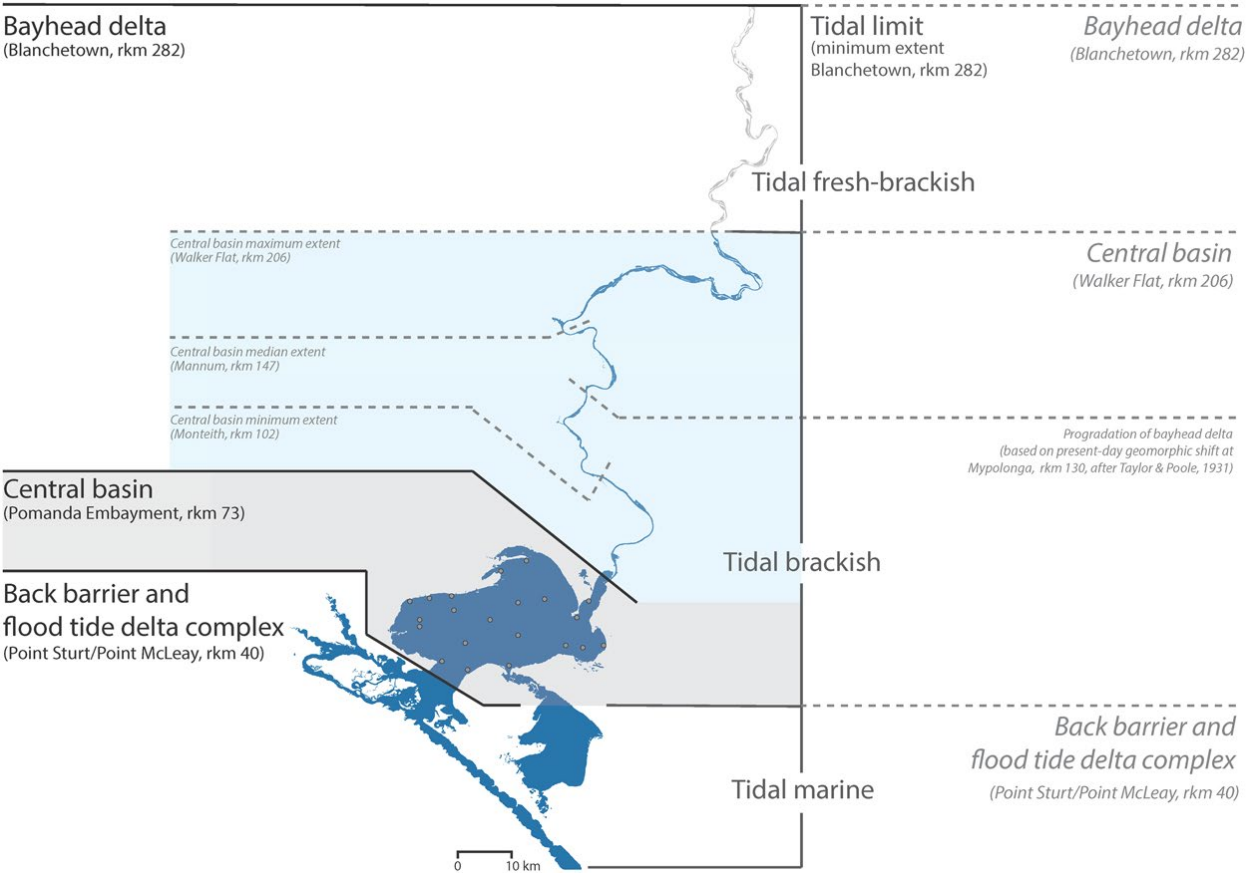
Estuarine processes zonation and inferred resulting morphology at the Holocene highstand. Based on S_mid_WL_2_ scenarios, estuarine processes can be categorised into three zonations: tidal marine, tidal brackish and tidal fresh-brackish (grey text). At the Holocene highstand, the tidal limit propagated beyond the study region, suggesting a minimum tidal limit of Blanchetown (rkm 282). This zonation is extrapolated into inferred resulting morphology at the Holocene highstand (grey italicised text). The Murray estuary’s barrier and flood tide delta complex occupied the region seaward of Point Sturt and Point McLeay (rkm 40). The central basin occupied the entirety of the Lower Lakes, Lakes Alexandrina and Albert, and extended upstream within the Murray Gorge plausibly as far as Walker Flat (rkm 206, minimum Monteith at rkm 102, and median Mannum at rkm 147). Upstream, the bayhead delta occupied a low-energy backwater zone at least as far as Blanchetown (rkm 282). During the late-Holocene, the bayhead delta prograded downstream to Mypolonga (rkm 130). Our results contrast conventional barrier estuary facies models (black text), which place the upstream limit of the central basin at the point where the river debouches into the lake or lagoon (i.e., Pomanda Embayment, rkm 73). The Murray estuary’s laminated central basin deposits have previously been identified in sediment cores (grey points) taken from within the conventional limits of the central basin (grey shaded area)^23,24^. Our results suggest that this laminated sequence characterises the Holocene depositional fill within the Murray Gorge at least as far upstream as Monteith (rkm 102) and plausibly as far as Walker Flat (rkm 206; grey and blue shaded areas).

Overall, the S_low_WL_2_ and S_mid_WL_2_ scenarios are well constrained by the Malcolm soil combination, and palaeo-shorelines, representing the maximum Holocene inundation extent of the Lower Lakes^31^ which suggests the suitability of interpolating these results to the palaeo-environment at the Holocene highstand (Fig. 2; Table S1). These results show that the Holocene highstand probably generated valley-wide inundation within the entirety of the Murray Gorge at least as far upstream as Blanchetown (rkm 282; Fig. 2), which coincides with the minimum propagation of the tidal limit of the Murray estuary (Fig. 6). Given this single central basin depositional environment, we infer the presence of a laminated sequence within the valley-wide LMR perhaps extending as far upstream as Walker Flat (rkm 206; Fig. 6), a hypothesis that will be tested by a complementary sedimentary analysis in a subsequent study. During the late-Holocene, we suggest that the bayhead delta prograded downstream to Mypolonga (rkm 130), where there is a notable shift in the geomorphic character of the levees and fringing swamps, before anthropogenic modification inhibited further natural estuarine evolution from 1900 onwards^32^.

### Sensitivity testing

Examples of the influence of bathymetric surface on maximum palaeo-salinities are shown in Figs 3 and 4a and on inundation extent in Fig. 2. The LMR is characterised by a main channel with fringing swamps under S_up_ scenarios, while the S_mid_ and S_low_ scenarios exhibit valley-wide inundation (Fig. 2; Table S1). Within the Lower Lakes, the modelled inundation extent is comparable to present-day shorelines under S_up_ scenarios, with the S_mid_ and S_low_ morphologies extending inundation across the Cooke Plains Embayment (Fig. 2; Table S1). Variation in palaeo-salinities is facilitated by Lake Alexandrina’s palaeo-channel within the S_mid_ and S_low_ morphologies, forcing marine influence upstream, pushing the brackish limit well within the Murray Gorge and causing the majority of the LMR to be characterised by brackish-fresh water (Figs 3a, 4a and S1b–c,e–f; Table S1). By comparison, the S_up_ scenarios allow for a brackish-fresh channel within the LMR while the fringing swamps largely remain fresh (Figs 3a, 4a and S1a,d; Table S1). The presence, or infill of, this palaeo-channel also alters the palaeo-salinity of Lake Albert (Figs 3a and S1). Overall, however, variation in bathymetric surface alone is insufficient to alter the palaeo-environment of the region, as demonstrated by comparing the results presented in Figs 3a and 4a. Despite the uncertainty in the precise location of a S_mid_ surface, the similarity between the results from the two morphological end members (S_low_ and S_up_ scenarios) show that robust conclusions can be drawn irrespective of the validity of the S_mid_ Holocene highstand best-estimate morphology.

Variation in barrier morphology exerts its greatest influence within the barrier and flood tide delta complex and attenuates rapidly; by the mid-section of the Lower Lakes the impact of barrier morphology is negligible (Figs 3c and 4a). The variety of postulated early- to mid-Holocene chain-of-islands evolution events in the barrier complex does not change the character of the palaeo-environment, as demonstrated by the near identical maximum palaeo-salinities and 10 psu (marine-brackish) limits presented in Figs 3c and 4a respectively.

Sensitivity testing for discharge reveals that the flood event has a greater influence on palaeo-salinities when compared to drought, however, only under S_up_WL_0_ scenarios is a flood sufficient to supress Lake Alexandrina to fresher conditions (Fig. 4a; Table S1). Drought conditions have a more pronounced impact on palaeo-salinities under present-day sea levels (WL_0_) when compared to WL_2_ scenarios (Fig. 4a). Overall, variation of discharge has a moderate influence on palaeo-salinities throughout the region, however, as demonstrated by the near-identical maximum palaeo-salinities and 10 psu (marine-brackish) limits presented in Figs 3b and 4a, discharge alone is unable to alter the palaeo-environmental character of the region.

## Discussion

Here, we assess the palaeo-Murray estuary’s response to the Holocene highstand exploring the hydrologic, hydrodynamic and geomorphic influences on the regional palaeo-environment. The experimental hydrodynamic modelling approach adopted in this study allows for the relative importance of drivers of palaeo-environmental change to be determined. Sensitivity testing for sea level, discharge, bathymetry and barrier morphology indicates sea level to be the determining factor for environmental characterisation of the palaeo-Murray estuary and the probable primary driver of change during the region’s Holocene evolution. The experimental hydrodynamic modelling approach used here subjects end-member conditions to a sensitivity analysis giving a range of plausible responses rather than an explicit replication of reality. For instance, the S_low_ (Pleistocene-Holocene boundary) surface is certainly deeper than reality at the Holocene highstand, and the S_up_ (pre-regulation) surface certainly shallower. The negligible difference in results obtained through this end-member approach signifies that our models can in fact be extrapolated to represent a reasonable approximation of reality at the Holocene highstand.

The modelled estuarine environment at highstand is well constrained by global-scale estuary initiation at 8,200 yr BP following a significant meltwater pulse from the Laurentian ice sheet^34^. This event triggered major coastal change worldwide as the resulting accelerated rise in sea level caused a landward ‘jump’ in the estuarine zone^34^. On a local scale, within the Lower Lakes, flood tide delta and barrier complex stratigraphic^23,31,35^, diatom^23,36^ and midden analyses^37^ support the designation of the regional palaeo-environment as estuarine at the Holocene highstand. However, our results demonstrate that the estuarine palaeo-environment was not limited solely to this region. We show that the +2 m Holocene highstand drove the estuarine limit much further upstream causing an enlarged low-energy backwater setting that occupied much of the LMR (minimum tidal limit rkm 282; Fig. 6). The low relief of this coastal plain facilitated an elongated central basin within the confines of the Murray Gorge, likely extending as far upstream as Walker Flat (rkm 206), where the silt-clay laminated sequence that characterises the central basin deposits within Lake Alexandrina^23,24^ are inferred to extend (Fig. 6). Adopting Zaitlin *et al*.’s^38^ nomenclature, our results demonstrate that the middle incised valley extends from the modern-day shoreline landward to the estuarine limit at highstand, which we place between Walker Flat (rkm 206) and Blanchetown (rkm 282; Figs 4b, 6 and S2). We suggest that, at highstand, the fluvial inner incised valley stretched from this estuarine limit landward to Overland Corner (rkm 439) where the river enters the Murray Gorge. Here, the Coonambidgal Formation displays evidence of a declining energy gradient^39^, which is not characteristic of the sediments of a meandering river 430 rkm upstream of its terminus. This unusually extensive backwater zone was a consequence of the unique low relief of this coastal plain system that attenuated flow velocities and forced the limit of coarse-grained fluvial deposition well upstream.

Since European settlement, the region has been subject to significant modification including extensive land reclamation and the construction of levees, locks, and barrages. Prior to this, the riverbanks between Mannum (rkm 154) and Mypolonga (rkm 130) were typically high creating natural levees that separated the channel from low-lying flats; this configuration transitioned to an unbroken series of swamps between Mypolonga (rkm 130) and Wellington (rkm 78)^32^. This shift in channel fringing environment at Mypolonga (rkm 130) is inferred to be the approximate limit of bayhead delta progradation before significant European modification and regulation of the LMR disrupted the natural flow regime (Fig. 6). A homogenous clay sequence could be expected to overlie the laminated central basin deposit, representing the downstream progradation of the bayhead delta, the precise location of which will be determined by a subsequent study.

The regionally extensive and continuous nature of the Murray estuary’s laminated deposit within Lake Alexandrina from 5,500 yr BP until modern-day^23,24^ suggests that the mechanism of deposition and preservation cannot exclusively be attributed to a palaeo-environment that differs to what we see today. The sequence continued to deposit despite the decrease in regional salinity brought about by a fall to present-day sea levels in the late-Holocene (Figs 3a and 4a). Recent flume studies demonstrate the capacity of laminated silt-clay deposition in much higher velocities than previously thought^26^, with our results illustrating a real-world application in a dynamic palaeo-environment subject to marine influence (Figs 5 and S3) and consistent with the notion that salinity assists, but is not vital, for floc formation and laminae deposition^25^. An Australian east coast analogue is present in the Hawkesbury River estuary, where the Holocene estuarine central basin and bayhead delta sediments extend well into the gorge-confined valley, with the Colo River estuarine sequence presenting similar laminations to those described in Lake Alexandrina^23,24,29,40^.

When considering the LMR, or other gorge confined portions of coastal plain estuaries as an extension of the central basin, the definition adopted is important. Dalrymple *et al*.^30^ define the central basin not in the geomorphic sense of a lagoon, with which gorge confined regions such as the LMR could not conceivably be considered, but rather on the basis of facies designation. We correlate the process-based results from S_mid_WL_2_ scenarios with facies designation to infer the resulting morphology of the Murray estuary at the Holocene highstand (Fig. 6). Our inferred resulting morphology adopts Dalrymple *et al*.’s^30^ facies rather than a geomorphic definition of the central basin whereby the lower portion of incised river valleys may exhibit the depositional characteristics of the central basin. Here we consider the central basin as the region of lowest energy characterised by the confluence of marine and fluvial influence and the deposition of the finest sediment.

Following conventional models of estuarine facies designation, the location where the river debouches into the lagoon is the likely transitional point of the designation of fluvial to estuarine geological formations (Fig. 6). However, with low relief allowing for elongated estuarine zones at the Holocene highstand, we suggest that the location of this transition requires review across coastal plain estuaries more broadly. In the case of the Murray estuary, the Holocene stratigraphy of Lake Alexandrina is characterised as the estuarine and coastal-marine sediments of the St Kilda Formation, whereas the LMR is characterised as the Quaternary alluvium of the Coonambidgal Formation (Fig. 2a; Table S2). This transition is currently placed at the Pomanda Embayment (rkm 73; Figs 1 and 2a), or precisely where the LMR debouches into Lake Alexandrina. By assigning our process-based results to inferred resulting morphology, we suggest a revision of the location of this transition is required to account for the Holocene extension of estuarine sedimentation within the gorge-confined portion of the central basin (Fig. 6). A sedimentary analysis is currently underway to assess the upstream extent and nature of this deposit, with previous work in the region suggesting the presence of a laminated sequence may be widespread within the LMR^32,41^.

The key to understanding responses of coastal plain estuaries to future changes in climate requires a knowledge of drivers of change, best explored by an examination of palaeo-responses to such change through representative analogues. Here we demonstrate the vulnerability of Australia’s largest and most politically and economically significant river basin to future environmental change. A comparison between S_up_WL_0_ and S_up_WL_2_ scenarios reveal the pronounced shift in environmental character with higher sea levels inducing significant marine incursion to the Lower Lakes and driving the brackish and fresh-brackish water zones as far upstream as Teal Flat (rkm 183; Figs 3a I,IV and S1a,d). Currently, the Murray estuary is a highly regulated system with a series of barrages in place to prevent saline intrusion into the estuary and river system, crucial for maintaining the freshwater resources within the region during ‘undesirable’ weather events such as prolonged drought. With the pace of future sea-level rise too rapid for barriers to transgress in response, and our results demonstrating a significant change in environmental character regardless of barrier morphology, we demonstrate the utility of applying a historical analogue to understand the importance of adapting water management to future needs. In the case of the Murray estuary, this analysis highlights the future importance of and likely need for reliance on the barrages if the current freshwater resource priorities are to be maintained.

Adopting a hydrodynamic modelling approach to Holocene analogues of coastal plain estuaries allows for the significant potential impact of climate change induced sea-level rise to be realised. Our results identify sea level as the dominant controlling factor on the environmental character of the Murray estuary, with the approximately 2 m higher-than-present sea level during the Holocene highstand generating an extensive central basin environment characterised by a low-energy backwater and laminated silt-clay deposits. We demonstrate that the estuarine limit can extend significantly further inland than expected when evaluating modern-day geomorphology in the context of conventional estuarine facies models. The importance of sea level in controlling the character of the Murray estuary, irrespective of fluvial discharge, bathymetry and barrier morphology, suggests the impacts of future sea-level rise due to climate change on coastal plain estuaries may be underappreciated. Our results are broadly applicable to low-gradient coastal plain estuaries with wave-dominated entrances, particularly those with large catchments and low discharges. However, consideration must be applied to the nature of the incised valley and valley fill, dynamics of fluvial discharge and tidal regime, as well as the rate of sea-level rise/fall when transferring these results to other coastal plain estuaries^6^. The extent and impact of sea-level rise as a driver of environmental change is largely a consequence of the inherently low gradient of these systems. This characteristic low gradient of coastal plain estuaries facilitates the landward extension of the estuarine zone rendering lower portions of the conventionally fluvially dominated zone particularly vulnerable to saline intrusion and potentially unable to support potable water or irrigation supplies. The economic and social implications of our findings to the LMR and Murray estuary, and comparable coastal plain estuaries more broadly, are considerable.

## Methodology

### Overview of model result categories

The study area encompasses the LMR from Blanchetown (rkm 282) downstream to the barrier complex and modern-day Murray Mouth (Fig. 1). Using TUFLOW FV, a 2D finite volume numerical model, we simulate 72 scenarios and conduct sensitivity testing for bathymetric surface (two end members and a best estimate), sea level (Holocene highstand and present-day), discharge (drought, pre-regulation average, and flood), and barrier morphology (four scenarios, ranging from completely open to almost closed, to account for barrier evolution). Results are grouped into six categories based on bathymetric surface and sea level (Table S1). The Pleistocene-Holocene stratigraphic boundary and pre-regulation surfaces represent bathymetric end-members to constrain the entire range of plausible bathymetries at the Holocene highstand. These are denoted as S_low_ and S_up_ respectively. A best estimate of bathymetry at the Holocene highstand is given by the S_mid_ surface. Accounting for the approximately 2 m variance in sea level between the Holocene highstand and present day gives the six modelled categories: S_low_WL_2_, S_low_WL_0_, S_mid_WL_2_, S_mid_WL_0_, S_up_WL_2_ and S_up_WL_0_ (Table S1). For each of these six categories, all possible combinations of discharge and barrier morphology were modelled. The three discharge scenarios of drought, pre-regulation average and flood are denoted by D_-_, D_av_ and D_+_ respectively (Table S1). The four barrier morphologies of completely open, two evolutionary phases, and modern-day are denoted by B_0_, B_+_, B_++_ and B_mod_ respectively (Fig. 1c–f; Table S1). We obtain inundation extents, water heights and depths, flow velocities and salinities for the full extent of the LMR and Murray estuary for each of the 72 modelled scenarios.

### Numerical model set up

Hydrology is simulated using TUFLOW FV, a 2D finite volume numerical model. The model domain spans some 282 rkm from Lock 1 at Blanchetown to the Murray Mouth and extending 2 km offshore. A base model was provided by BMT WBM and was the subject of vigorous calibration^42^ (Supplementary methods: Model calibration). Stitched topography and bathymetry for the region was developed by the Commonwealth Scientific and Industrial Research Organisation (CSIRO)^43^ and provided by the South Australian Department of Environment Water and Natural Resources (DEWNR) for use in this study. Outside the extent of this dataset (1956 flood extent), a 1 second Digital Elevation Model (DEM), provided by Geoscience Australia (GA), was applied and the two datasets interpolated together using ArcGIS. This mesh was then modified to extend the model domain to encompass the entire width of the Murray Gorge, as well as the inclusion of the modern-day barrier complex and extension of the Lower Lakes based on the palaeo-maximum inundation shoreline and Holocene estuarine stratigraphy^31,35,44^. Tides were imposed based on historical data taken from Victor Harbour between 1^st^ January – 28^th^ February 2014 to remove the uncertainties associated with tidal prediction (Fig. S4). Drought (D_-_) and pre-regulation average (D_av_) scenarios were run for 20 days while flood (D_+_) scenarios were run for 31 days, which was a sufficient period for models to run beyond the spin-up phase and reach steady state, as confirmed by a review of hydrograph phasing. All models were run at a 5 minute timestep. A comparative analysis of 24 and 1 hour outputs confirmed that the 24 hour outputs were representative and, to save computational time, were subsequently applied to all scenarios. Initial salinity was applied at each cell based on salinity data taken from 25 gauging stations throughout the region at the peak of the Millennium drought. This was deemed appropriate as the barrages are in place to curtail saline intrusion and therefore regional salinities are held fresher than would naturally occur. Due to the potential influence of stratification of the estuary a representative subset of models were run in 3D to assess the suitability of adopting computationally efficient 2D models for this study. Refer to Supplementary methods: Comparison of 2D and 3D simulations for further information. The bottom drag model adopted for this study is the Manning’s coefficient, with a global value of 0.025 applied to the entire model domain. This value is supported by sensitivity testing and calibration performed by BMT WBM on the base model provided for this study^42^, and aligns with sensible values given hypothesised Holocene regional palaeo-environmental conditions^45^. Refer to Supplementary methods: Model calibration for further information.

### Morphology

To best resolve bathymetry and topography at highstand, three surfaces were created: (1) a pre-regulation surface (S_up_) provided a modern-day end member, (2) the depth to the Monoman – Coonambidgal Formation transition provided a Late Pleistocene – Early Holocene end member (S_low_), and (3) a best estimate of highstand bathymetry and topography (S_mid_). Details on the creation of the three bathymetric surfaces, and chain-of-islands evolution of Sir Richard and Younghusband Peninsulas used to inform the four modelled barrier morphologies are given in Supplementary methods: Morphology.

### Sensitivity testing

Sensitivity testing for barrier evolution was based on the chain-of-islands model^13^ with the location of possible palaeo-outlets interpreted from Bourman and Murray-Wallace^46^, de Mooy^31^ and Luebbers^37^. Four barrier configurations were tested ranging from the complete removal of Sir Richard and Younghusband Peninsulas to the modern-day Murray Mouth (Fig. 1c–f; Table S1). Three discharge conditions were tested: two held constant at the Millennium drought low flow (D_−_; 152 m^3^/sec) and pre-regulation average flow (D_av_; 419 m^3^/sec), and one variable to simulate a flood, with pre-regulation average discharge (D_av_) increasing to the peak of the 1974 flood (D_+_; 1,883 m^3^/sec) and decreasing again^47,48^ (Table S1).

There have been numerous sea-level studies across Australia, with the majority stemming from east coast datasets^19,49^. As a consequence of isostatic and climatic influences, and localised geomorphology, there is wide variability across the Australian coast in both the magnitude and timing of the Holocene sea-level highstand^19^. Highstand estimates must therefore be derived from the regional setting of the study area which, for the Murray estuary, limits data to studies from the Gulf of St Vincent and the Spencer Gulf in South Australia. Immediately prior to the highstand (8,000–7,500 yr BP), sea level reached present day levels^19,20,50^ with the magnitude of the subsequent highstand ranging from +1 m up to +3 m across the southern Australian coast^19,20^. This study adopts a best approximation of a +2 m highstand at 7,000–6,000 yr BP^19,20^, a value which has been adopted by other studies of the Holocene palaeo-Murray estuary^18^. Our models were run twice – once using present day tides (WL_0_) and again at present day tides plus 2 m to simulate Holocene highstand conditions (WL_2_; Table S1).

### Post-processing

Salinity was classified based on chloride concentration using Tooley’s^51^ scheme: fresh <0.1 g Cl/L, fresh-brackish 0.1–0.5 g Cl/L, brackish-fresh 0.5–1 g Cl/L, brackish 1–5 g Cl/L, brackish-marine 5–10 g Cl/L, marine-brackish 10–17 g Cl/L, and marine >17 g Cl/L. Salinity was assessed using maximum salinities observed post model burn-in phase. We considered maximum rather than average salinity as, due to constraints in computational power giving a 5-fold increase in model run time, salinity is not resolved in 3D therefore results do not account for a salt wedge at depth but rather depict a freshwater plume at the surface. Directional vectors were assessed within the present-day channel (and not fringing swamps) such that a direct comparison could be drawn between the three model surfaces regardless of inundation extent or bathymetrically-controlled primary flow path. Velocity magnitude was considered relative to the critical threshold of 0.3 m/s^25–27^ and representative models were re-run to assess tidal signatures from water levels at 1 hour outputs.

## Supplementary information

Supplementary Materials

## References

[1] Rogers K, Woodroffe CD (2016). Geomorphology as an indicator of the biophysical vulnerability of estuaries to coastal and flood hazards in a changing climate. J. Coast. Conserv..

[2] Reisinger, A. *et al*. In *Climate Change 2014: Impacts, Adaptation, and Vulnerability. Part B: Regional Aspects*. [Barros, V. R. *et al*., (eds)] Contribution of Working Group II to the Fifth Assessment Report of the Intergovernmental Panel on Climate Change pp. 1371–1438 (Cambridge University Press 2014).

[3] Baynes, T., Herr, A., Langston, A. & Schandl, H. Coastal Climate Risk Project Milestone 1 Final Report to the Australian Department of Climate Change and Energy Efficiency. Prepared for the Department of Climate Change and Energy Efficiency (DCCEE) by the Commonwealth Scientific and Industrial Research Organisation (CSIRO) (DCCEE 2012).

[4] Woodroffe CD, Murray-Wallace CV (2012). Sea-level rise and coastal change: the past as a guide to the future. Quat. Sci. Rev..

[5] Blum MD, Törnqvist TE (2000). Fluvial responses to climate and sea-level change: a review and look forward. Sedimentology.

[6] Roy PS, Thom BG, Wright LD (1980). Holocene sequences on an embayed high-energy coast: an evolutionary model. Sediment. Geol..

[7] Sloss, C. R., Murray-Wallace, C. V. & Jones, B. G., Aminostratigraphy of Two Holocene Wave-Dominated Barrier Estuaries in Southeastern Australia. *J. Coast. Res*., 113–136 (2006).

[8] Sloss CR, Jones BG, McClennen CE, De Carli J, Price DM (2006). Mid- to late Holocene sedimentation in a coastal lagoon: Burrill Lake, NSW, Australia. Journal of Sedimentary Geology.

[9] Sloss CR (2010). The Holocene infill of Lake Conjola, a narrow incised valley system on the southeast coast of Australia. Quat. Int..

[10] Thom BG, Roy PS (1985). Relative sea-levels and coastal sedimentation in southeast Australia in the Holocene. J. Sediment. Petrol..

[11] Umitsu M, Buman M, Kawase K, Woodroffe CD (2001). Holocene palaeoecology and formation of the Shoalhaven River deltaic-estuarine plains, southeast Australia. The Holocene.

[12] Cann JH, Murray-Wallace CV, Belperio AP, Brenchley AJ (1999). Evolution of Holocene coastal environments near Robe, southeastern South Australia. Quat. Int..

[13] Harvey, N. Holocene Coastal Evolution: Barriers, Beach Ridges, and Tidal Flats of South Australia. *J. Coast. Res*. 90–99 (2006).

[14] Belperio, A. P., Hails, J. R. & Gostin, V. A. A review of Holocene sea levels in South Australia, Occassional Paper 3. [Hopley, D. (ed.)] Australian sea levels in the last 15 000 years: a review (James Cook University 1983).

[15] Mills K (2013). Paleoclimate studies and natural-resource management in the Murray-Darling Basin I: past, present and future climates. Aust. J. Earth Sci..

[16] Hill PJ, De Deckker P, von der Borch C, Murray-Wallace CV (2009). Ancestral Murray River on the Lacepede Shelf, southern Australia: Late Quaternary migrations of a major river outlet and strandline development. Aust. J. Earth Sci..

[17] Fluin, J., Haynes, D. & Tibby, J. An Environmental History of the Lower Lakes and the Coorong. Report commissioned by the South Australian Department of Envrionment and Heritage, Adelaide (2009).

[18] Bourman RP, Murray-Wallace CV, Belperio AP, Harvey N (2000). Rapid coastal geomorphic change in the River Murray Estuary of Australia. Mar. Geol..

[19] Lewis SE, Sloss CR, Murray-Wallace CV, Woodroffe CD, Smithers SG (2013). Post-glacial sea-level changes around the Australian margin: a review. Quat. Sci. Rev..

[20] Belperio AP, Harvey N, Bourman RP (2002). Spatial and temporal variability in the Holocene sea-level record of the South Australian coastline. Sediment. Geol..

[21] Harvey N, Belperio A, Bourman R, Mitchell W (2002). Geologic, isostatic and anthropogenic signals affecting sea level records at tide gauge sites in southern Australia. Global Planet. Change.

[22] Twidale, C. R., Lindsay, J. M. & Bourne, J. A. Age and origin of the Murray River and Gorge in South Australia [Warren, J. W. (ed.)] Proceedings of Royal Society of Victoria, pp. 27–42 (The Royal Society of Victoria 1978).

[23] Barnett, E. J. Recent Sedimentary History of Lake Alexandrina and the Murray Estuary. The Flinders University of South Australia (1993).

[24] Barnett EJ (1994). A Holocene paleoenvironmental history of Lake Alexandrina, South Australia. J Paleolimnol.

[25] Schieber J, Yawar Z (2009). A new twist on mud deposition - mud ripples in experiment and rock record. The Sedimentary Record.

[26] Schieber J, Southard J, Thaisen K (2007). Accretion of Mudstone Beds from Migrating Floccule Ripples. Science.

[27] Baas JH, Best JL, Peakall J (2016). Predicting bedforms and primary current stratification in cohesive mixtures of mud and sand. J. Geol. Soc..

[28] Allen JRL (2004). Annual textural banding in Holocene estuarine silts, Severn Estuary Levels (SW Britain): patterns, cause and implications. The Holocene.

[29] Devoy RJ, Dodson JR, Thom BG, Nichol S (1994). Holocene environments in the Hawkesbury valley, new South Wales: A comparison of terrestrial and marine records. Quat. Sci. Rev..

[30] Dalrymple RW, Zaitlin BA, Boyd R (1992). Estuarine facies models; conceptual basis and stratigraphic implications. J. Sediment. Res..

[31] de Mooy CJ (1959). Notes on the geomorphic history of the area surrounding Lakes Alexandrina and Albert. Trans. Roy. Soc. S. Aust..

[32] Taylor, J. K. & Poole, H. G. A soil survey of the swamps of the lower Murray River, S. Council for, R. Industrial, Eds., Bulletin (Council for Scientific and Industrial Research (Australia)); no. 51. (C.S.I.R, Melbourne 1931).

[33] Sloss CR, Jones BG, McClennen CE, de Carli J, Price DM (2006). The geomorphological evolution of a wave-dominated barrier estuary: Burrill Lake, New South Wales, Australia. Sediment. Geol..

[34] Rodriguez AB, Simms AR, Anderson JB (2010). Bay-head deltas across the northern Gulf of Mexico back step in response to the 8.2 ka cooling event. Quat. Sci. Rev..

[35] Von der Borch C, Altmann M (1979). Holocene stratigraphy and evolution of the Cooke Plains Embayment, a former extension of Lake Alexandrina, South Australia. Trans. Roy. Soc. S. Aust..

[36] Cann JH, Bourman RP, Barnett EJ (2000). Holocene Foraminifera as Indicators of Relative Estuarine-Lagoonal and Oceanic Influences in Estuarine Sediments of the River Murray, South Australia. Quat. Res..

[37] Luebbers, R. A. The Coorong Report: An archaeological survey of the Northern Coorong, Prepared for the South Australian Department for Environment and Planning (1982).

[38] Zaitlin, B. A., Dalrymple, R. W. & Boyd, R. The stratigraphic organization of incised-valley systems associated with relative sea-level change, [Dalrymple, R. W., Boyd, R. & Zaitlin, B. A., (eds)] Incised-Valley Systems: Origin and Sedimentary Sequences, Special Publication No. 51, pp. 45–60 (SEPM (Society for Sedimentary Geology), Tulsa, Oklahoma, U.S.A. 1994).

[39] De Carli, E. & Hubble, T. C. T. Morphological characteristics of riverbank failure on the lower River Murray, South Australia, [Vietz, G., Rutherfurd, I. D. & Hughes, R., (eds)] Proceedings of the 7th Australian Stream Management Conference, pp. 255–261 (Townsville, Queensland 2014).

[40] Nichol SL, Zaitlin BA, Thom BG (1997). The upper Hawkesbury River, New South Wales, Australia: a Holocene example of an estuarine bayhead delta. Sedimentology.

[41] Hubble, T. C. T. & De Carli, E. Mechanisms and Processes of the Millennium Drought River Bank Failures: Lower Murray River, South Australia, *Goyder Institute for Water Research Technical Report* (Adelaide, South Australia, 2015).

[42] Hudson, R. Modelling Investigation into the Wellington ‘Virtual Weir’ Concept: Project summary report, BMT WBM, Prepared for the Murray Darling Basin Authority (MDBA) (MDBA 2010).

[43] Austin, J. M. & Gallant, J. C. Stitching elevation and bathymetry data for the Murray River and Lower Lakes, South Australia, CSIRO: Water for a Healthy Country National Research Flagship (CSIRO 2010).

[44] Murray-Wallace CV (2010). Aminostratigraphy and thermoluminescence dating of coastal aeolianites and the later Quaternary history of a failed delta: The River Murray mouth region, South Australia. Quat. Geochronol..

[45] Ladson, A., Lang, S., Anderson, B. & Rutherfurd, I. D. An Australian Handbook of Stream Roughness Coefficients, paper presented at the 28th Hydrology and Water Resources Symposium, Wollongong, 10–14 November (2003).

[46] Bourman B, Murray-Wallace CV (1991). Holocene evolution of a sand spit at the mouth of a large river system: Sir Richard Peninsular and the MurrayMouth, South Australia. Z. Geomorphol. Suppl..

[47] Bloss, C., Montazeri, M. & Eckert, G. Flood mapping of the River Murray floodplain in South Australia, Department of Environment, Water and Natural Resources (DEWNR) Technical Report 2015/57 (DEWNR 2015).

[48] Gippel, C. J. & Blackham, D. Review of environmental impacts of flow regulation and other water resource developments in the River Murray and Lower Darling River system, Final Report by Fluvial Systems Pty Ltd, Stockton, to Murray-Darling Basin Commission (MDBC), Canberra, ACT (MDBC 2002).

[49] Sloss CR, Murray-Wallace CV, Jones BG (2007). Holocene sea-level change on the southeast coast of Australia: a review. The Holocene.

[50] Bowman G, Harvey N (1986). Geomorphic Evolution of a Holocene Beach-Ridge Complex, LeFevre Peninsula, South Australia. J. Coast. Res..

[51] Tooley, M. J. Sea-level Changes: Northwest England during the Flandrian stage (Clarendon Press 1978).

