# Supplementary material for: Modelling Holocene analogues of coastal plain estuaries reveals the magnitude of sea-level threat
Source: Sci Rep. 2019 Feb 25;9:2667. doi: 10.1038/s41598-019-39516-4 (PMC6389980; doi:10.1038/s41598-019-39516-4)
Supplement: Supplementary file 1 — Supplementary Materials [file 41598_2019_39516_MOESM1_ESM.pdf]

SUPPLEMENTARY MATERIALS

Modelling Holocene analogues of coastal plain estuaries reveals the magnitude of sea-level threat

Authors: Anna M. Helfensdorfer[1,2]*, Hannah E. Power[2], Thomas C.T. Hubble[1]

Affiliations:

1. School of Geosciences, The University of Sydney, Sydney, NSW 2006, Australia

2. School of Environmental and Life Sciences, The University of Newcastle, Callaghan, NSW 2308, Australia

*Correspondence should be addressed to A.M.H. (email: anna.helfensdorfer@sydney.edu.au)

Supplementary methods: Model calibration

Following a significant period of prolonged drought, BMT WBM were commissioned by the Murray-Darling Basin Authority (MDBA) to perform a feasibility study assessing the adoption of a virtual weir at Wellington (rkm 78) to provide an adequate fresh water supply for the region. This involved the establishment, calibration and validation of a hydrodynamic model encompassing the LMR and Lower Lakes region downstream of Blanchetown (rkm 282, Lock 1). A validation dataset was collected, and the model calibrated for hydrodynamics - including water level, wind and river fluxes – and salinity. Model calibration adequately resolved short term (i.e. a single extreme saline intrusion event) and long term (i.e. entire 17 month dataset) trends.

Owing to the long-term temporal scale of modelling estuarine response to sea level change over the course of the mid- to late-Holocene, simplifications need to be applied based on best estimate assumptions to guide parameters for sensitivity testing. The model set up adopted in this study seeks to apply ‘appropriate complexity’ balancing a reductionist approach to input data based on geological correlation, to produce outputs which are computationally efficient yet meaningful (*1, 2)*. Model manipulation and model scenarios involved a deviation from present day morphology, flow and flow obstructions, sea level and ocean outlet, resulting in models which inherently could not be calibrated against the present day. Instead, results were compared to the Holocene stratigraphic record. Water heights were compared to documented evidence of notches and wave-cut cliffs along the former shoreline of Lake Alexandrina (*3*), and inundation extents correlated with the Malcolm soil combination and sediments of the Cooke Plains Embayment (*4, 5*).

Wave data was excluded from our model as the primary influence of waves within a wave-dominated estuary is as a driver of morphological change through the formation of a barrier complex at the estuary mouth and this model does not incorporate a sediment transport or morphology component (*6*). Furthermore, although this estuary is wave-dominated at its entrance, owing to the immense scale of this system, areas subject to significant wave energy present a negligible component of the overall model domain (Fig. S5).

A Manning’s coefficient of 0.025 was adopted across the model domain for this study. Applying a varying Manning’s coefficient was not actually implementable without a robust understanding of surficial sediments at the Holocene highstand over the entire 282 rkm of the model domain. Given this impracticality, applying a global Manning’s coefficient was deemed sensible and, although not an accurate representation of reality, this method nonetheless provides the means for direct comparison between results. The value of 0.025 was selected as it lies within the bounds of appropriate Manning’s coefficients given the likely Holocene palaeo-environmental conditions (*7*). Further, this value was deemed appropriate as sensitivity testing conducted during calibration of the base model provided by BMT WBM revealed that results did not vary significantly given changes in the Manning’s coefficient, but the model was best resolved when adopting values between 0.015 and 0.02538 (*8*). Reference 8 is a government funded study. For access, please contact the Murray Darling Basin Authority (MDBA) through: engagement@mdba.gov.au.

Supplementary methods: Morphology

To best assess the influence of morphology on the hydrodynamics of the system at the Holocene highstand, three surfaces were created. A pre-regulation surface (S_{up}) provided a modern-day end member, the depth to the Monoman – Coonambidgal Formation transition provided a late-Pleistocene –

early-Holocene end member (S_{low}), with the third surface a best estimate of highstand bathymetry and topography (S_{mid}). The S_{low} surface is certainly deeper than at highstand, with the average depth of the body of Lake Alexandrina (between Point Sturt/Point McLeay and Pomanda Embayment) approximately -43 m AHD (Australian Height Datum; approximately mean sea level). By comparison the S_{up} surface has an average of approximately -3 m AHD over the same area, with the S_{mid} best estimate highstand surface at approximately -8 m AHD. Within the LMR, the average of three surfaces is more closely constrained, varying from approximately -15 m to -8 m.

To resolve the S_{up} pre-regulation surface, the lock, barrages (and associated sediment sills), man-made levies and modern flood tide delta were removed from modern day DEMs (Fig. S6a). To resolve the S_{low} Pleistocene – Holocene surface, depths were interpreted from over 100 sediment cores, as well as interpretation of data and maps by Barnett (*9*) and Von der Borch and Altmann (*10*) (Fig. S6b; Table S3). The location and depth of the palaeo-channel within Lake Alexandrina was based on an interpretation of the work of Barnett (*9*) and geological maps (Fig. S6b). The S_{mid} best-estimate highstand surface has the greatest uncertainty as it was resolved by subtracting regional sedimentation rates from the pre-regulation surface (*9, 11*) and dated sediment cores (n = 18; Fig. S6c). Within the LMR and thalweg seaward, a sedimentation rate of 0.69 mm/y was adopted (*9, 11*). All other elements were adjusted with a sedimentation rate of 0.16 mm/y (*9, 11*).

The spatial interval of the three bathymetric surfaces created is identical to that of the modern-day input dataset as values were adjusted at each individual cell. The cell/element size varies considerably across the model domain with an average of approximately 50 m in cell side length within the LMR, modern flood tide delta (seaward of Point Sturt/Point McLeay, rkm 40) and Lower Lake fringes, and palaeo-Murray thalweg, with a considerably larger cell/element size within the main body of the Lower Lakes.

The chain-of-islands evolution of Sir Richard and Younghusband Peninsulas (*3, 4, 12, 13*) was the guiding premise for the series of barrier morphologies presented in this study. Bourman and Murray-Wallace (*13*) and de Mooy (*4*) give detailed descriptions and maps of hypothesised former outlets of the LMR to the ocean. These maps were georeferenced and digitised and assessed relative to the modern-day topography of the barrier system. Furthermore, the presence or absence of Aboriginal middens within the Holocene barrier (*3, 14, 15, 16*) were also mapped and assessed relative to their radiocarbon ages. These data were combined and analysed to produce a series of best estimates of the morphology of the mouth of the LMR as it evolved throughout the Holocene. As this study imposes a static barrier configuration for each scenario, an assessment of the dynamic response of the barrier to events on short temporal scales, such as tides or storms, was beyond the scope of the study.

Supplementary methods: Comparison of 2D and 3D simulations

Estuarine stratification can cause a salt wedge at depth which cannot be resolved by a 2D simulation. The presence of a salt wedge has the potential to alter inferences drawn from estuarine zonation and the likelihood of deposition of a laminated sequence whereby flocculation may be assisted by salinity. The scale of the study area, with the model domain spanning some 282 river kilometres, precluded the use of a 3D model setup without justification as the computational power to run such a simulation is ten times that of its 2D counterpart. Therefore, a representative subset of models were run in 3D to assess the suitability of adopting 2D models for this study. This representative subset of models allowed for a comparative assessment of 2D and 3D results for all bathymetric surfaces ($S_{low}WL_2D_{av}B_{mod}$, $S_{mid}WL_2D_{av}B_{mod}$ and $S_{up}WL_2D_{av}B_{mod}$), both sea level scenarios ($S_{mid}WL_2D_{av}B_{mod}$ and $S_{mid}WL_0D_{av}B_{mod}$), all discharge scenarios

($S_{mid}WL_2D_-B_{mod}$, $S_{mid}WL_2D_{av}B_{mod}$ and $S_{mid}WL_2D_+B_{mod}$) and all barrier morphologies ($S_{mid}WL_2D_{av}B_0$, $S_{mid}WL_2D_{av}B_{+}$, $S_{mid}WL_2D_{av}B_{++}$ and $S_{mid}WL_2D_{av}B_{mod}$).

3D simulations retained the same model setup with the inclusion of a parametric vertical mixing model with a second order vertical solution and density coupled salinity. A comparative analysis of results suggests that 2D simulations are sufficiently representative of 3D simulations (Fig. S7). Minor differences in maximum salinity reached across the region does not alter the designation of the palaeo-environment with 2D simulations providing a conservative approximation of 3D results (Fig. S7 a-b). Crucially, the brackish limit (equivalent to 1 psu) differed by a maximum of 1 rkm between 2D and 3D depth averaged results, with the exception of the pre-regulation (S_{up}) surface where the 3D simulation captured a salt wedge that penetrated 6 rkm further upstream ($S_{up}WL_2D_{av}B_{mod}$ scenario). A negligible change in the total area conducive to the deposition of a laminated sequence was also observed, with 2D simulations overestimating this area by an average of 4%, demonstrating that 2D approximations of 3D velocity magnitudes are appropriate (Fig. S7 c-d).

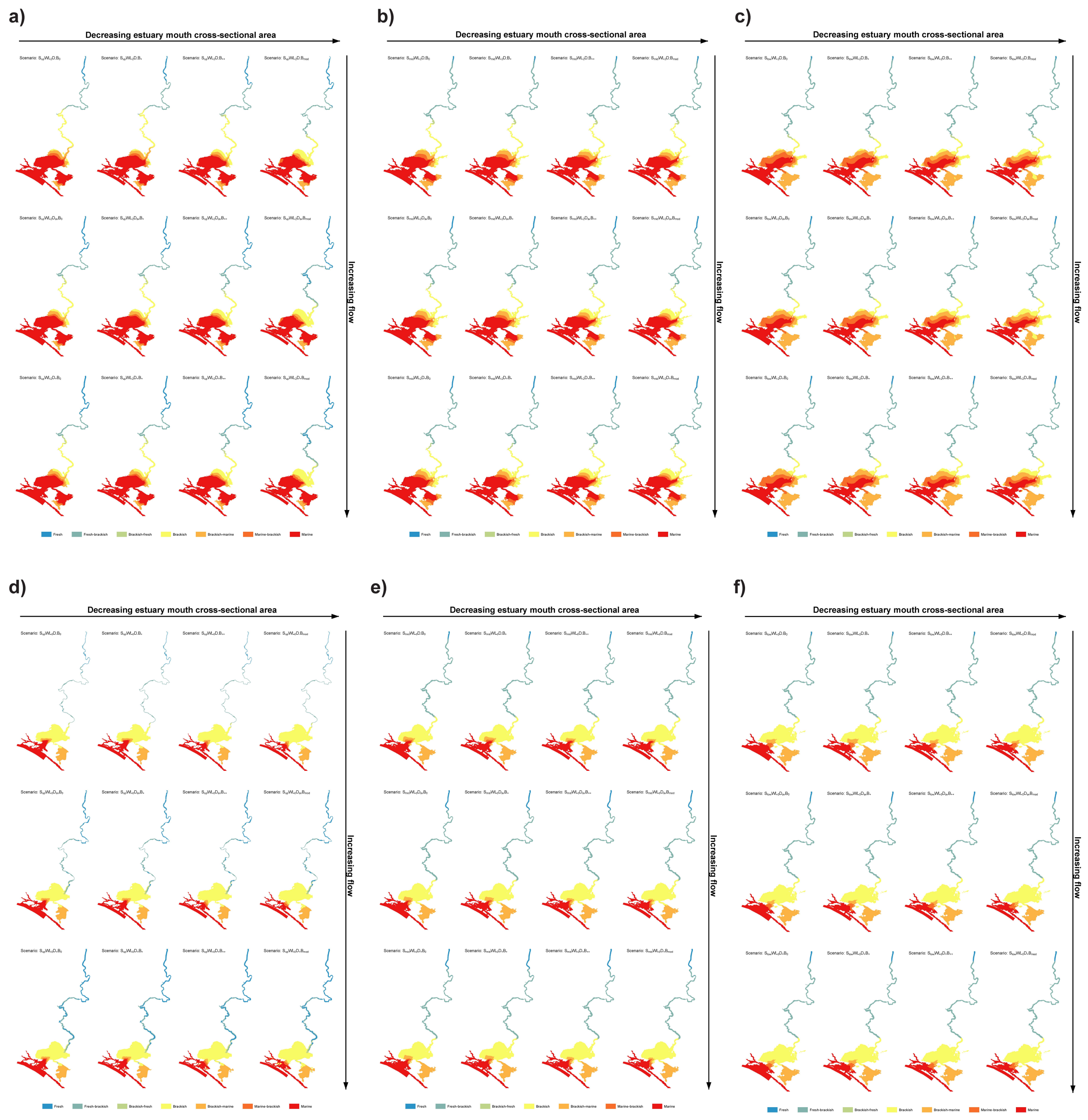

Figure S1: Maps of maximum salinity reached for each scenario. Maximum salinity is shown for each (**a**) $S_{up}WL_2$, (**b**) $S_{mid}WL_2$, (**c**) $S_{low}WL_2$, (**d**) $S_{up}WL_0$, (**e**) $S_{mid}WL_0$, (**f**) $S_{low}WL_0$ scenario. Salinity is measured based on the classification scheme of Tooley (*17*). Refer to Table S1 for scenario descriptions.

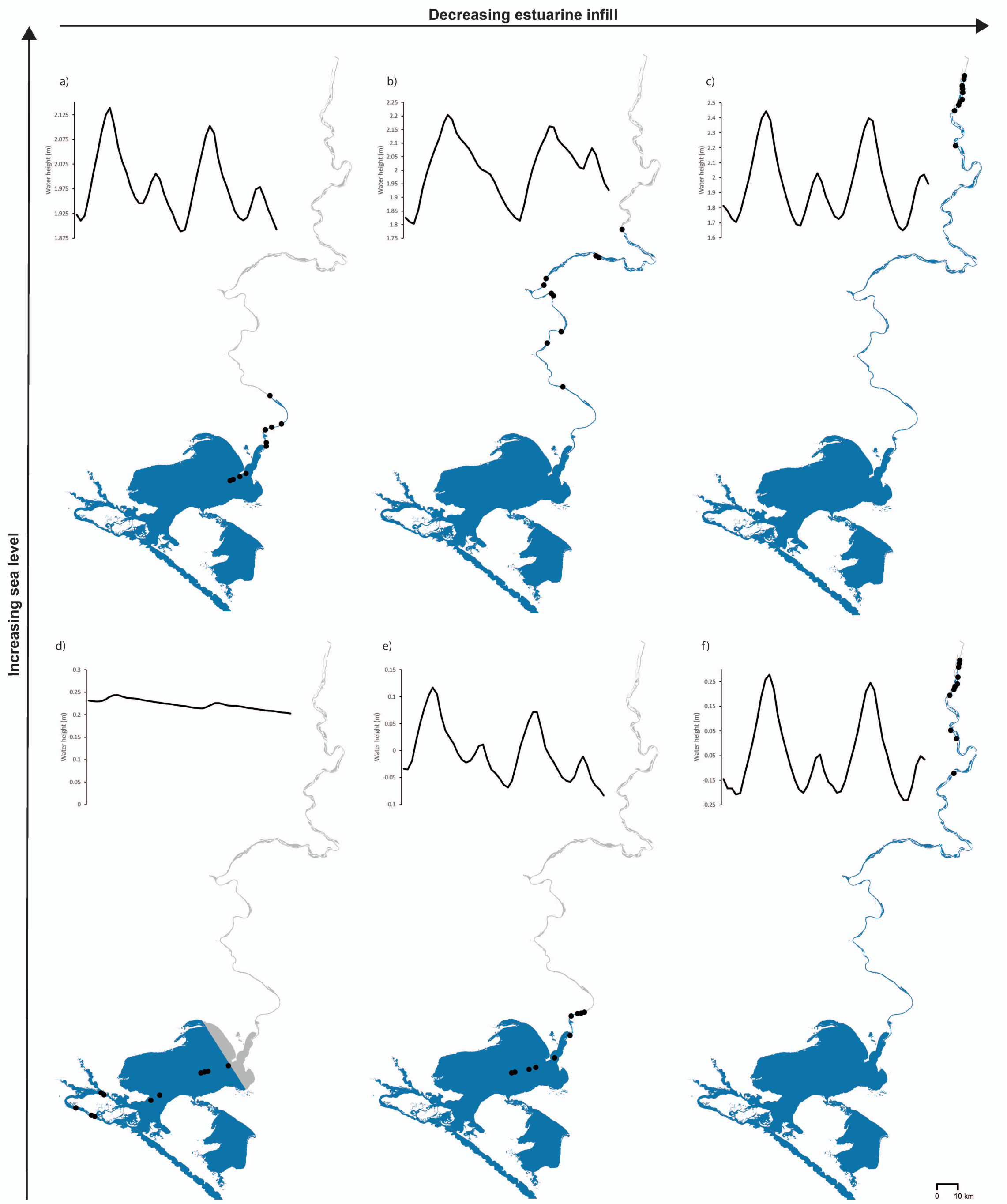

Figure S2: Characterisation of backwater zone and key representative tidal signatures. Maps show maximum upstream extent of velocity vector convergence for each scenario (black dots) in scenario category (**a**) $S_{up}WL_2$, (**b**) $S_{mid}WL_2$, (**c**) $S_{low}WL_2$, (**d**) $S_{up}WL_0$, (**e**) $S_{mid}WL_0$ and (**f**) $S_{low}WL_0$. Velocity vector convergence is taken as the point of convergence of upstream and downstream velocity vectors within the channel thalweg. Tidal signatures are given for the final 48 hours at the maximum upstream extent of upstream velocity vectors, in each scenario. Adopting Zaitlin's (*18*) nomenclature, the areas shaded in blue are characterised as the middle incised valley. At the Holocene highstand, an enlarged low-energy backwater setting was emplaced up to Walker Flat (**b**; rkm 206).

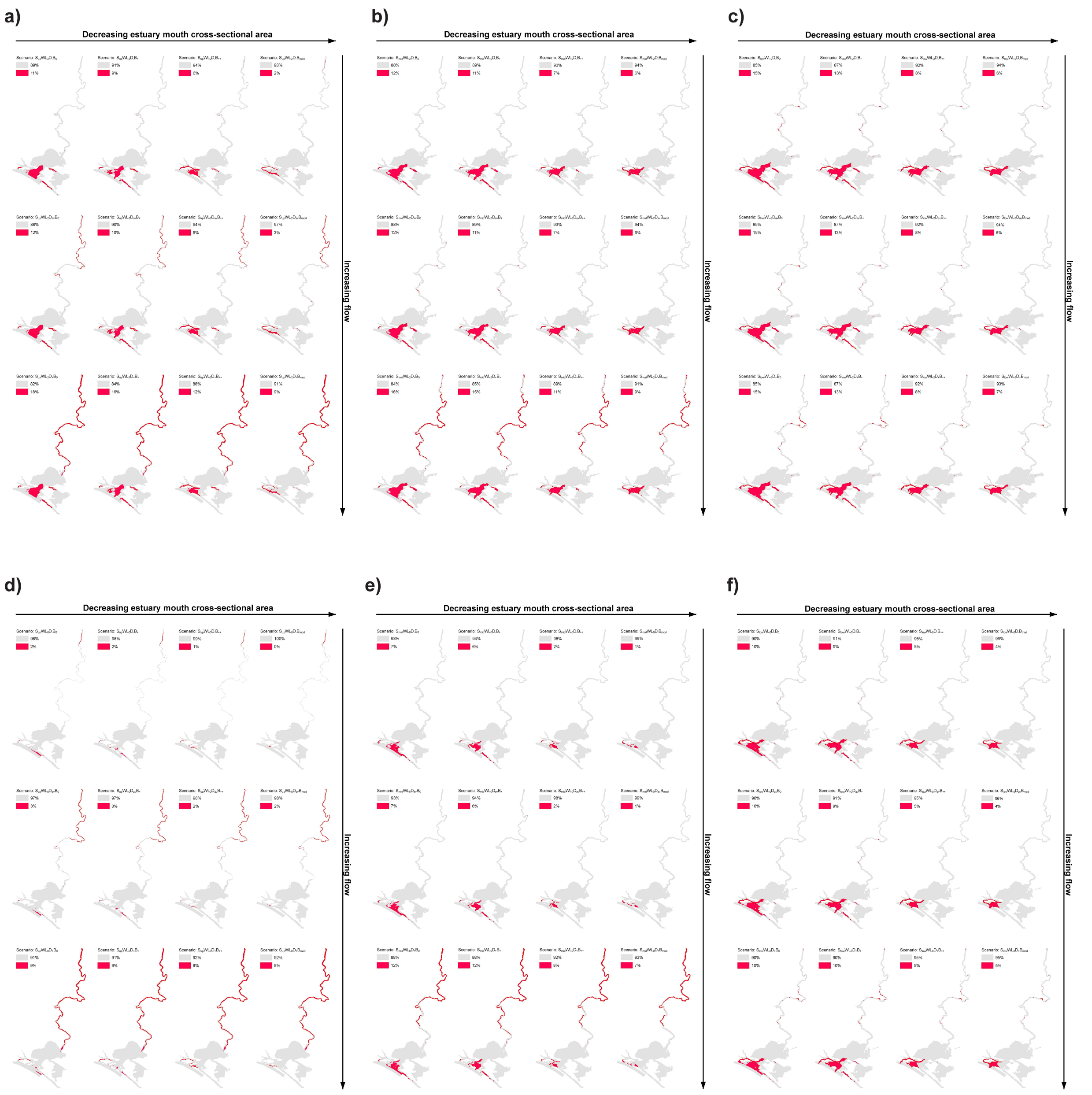

Figure S3: Maps of maximum velocity magnitude reached for each scenario. Maximum velocity magnitude is shown for each (**a**) $S_{up}WL_2$, (**b**) $S_{mid}WL_2$, (**c**) $S_{low}WL_2$, (**d**) $S_{up}WL_0$, (**e**) $S_{mid}WL_0$, (**f**) $S_{low}WL_0$ scenario. Areas are shaded red where maximum velocity > 0.3 m/s and therefore is not conducive to the deposition of a laminated silt-clay sequence (*19*, *20*, *21*). Refer to Table S1 for scenario descriptions.

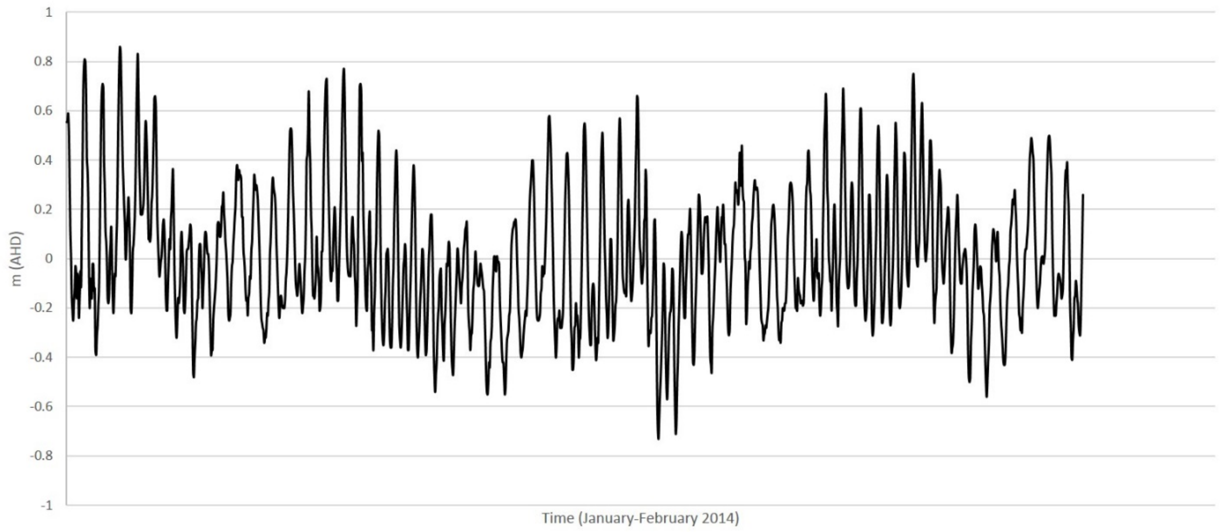

Figure S4: Tidal dataset adopted in this study. Data obtained from the Victor Harbour tidal gauge 01/01/2014 – 28/02/2014.

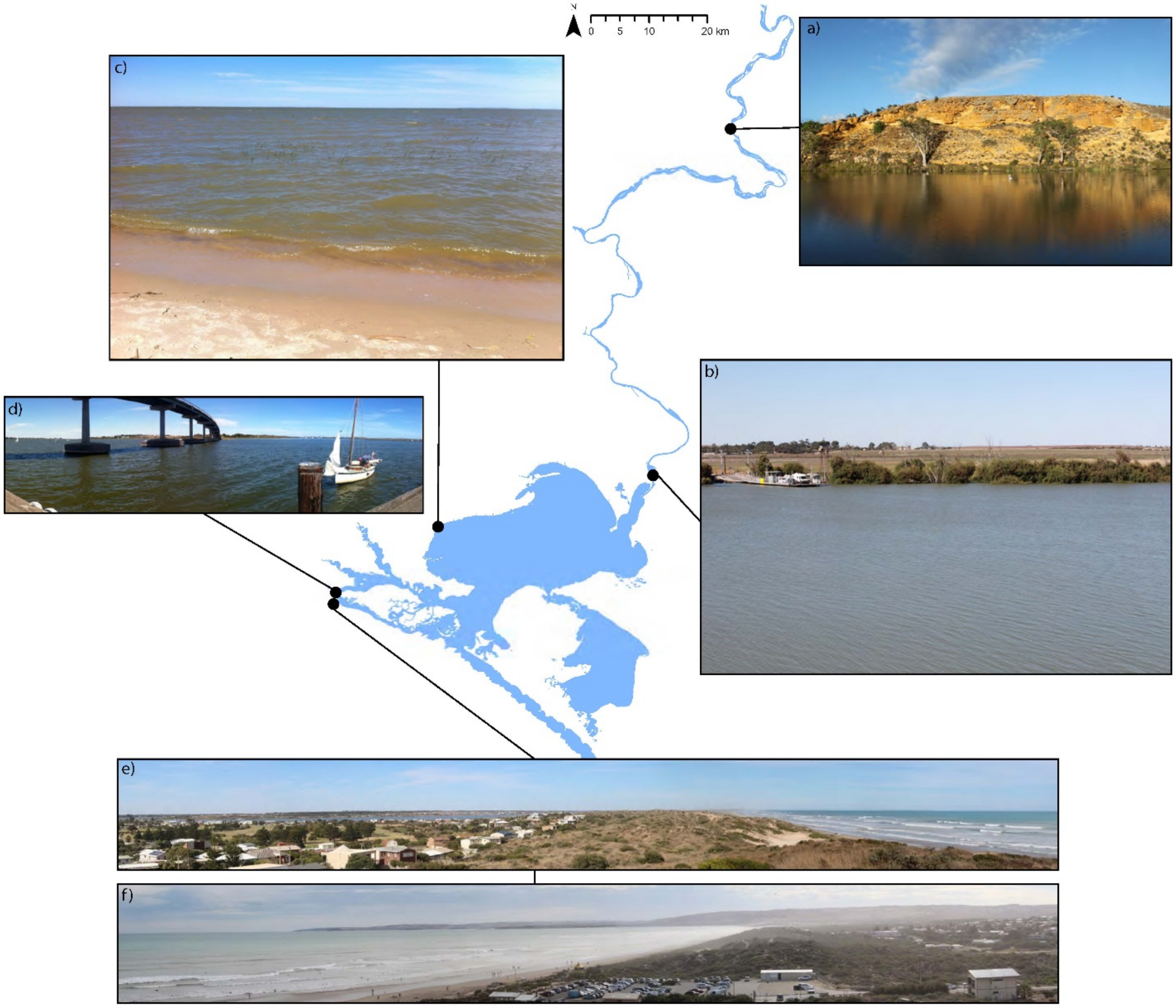

Figure S5: Overview map with site photos demonstrates the immense scale of the Murray estuary and lower Murray River. (**a**) The lower Murray River at Walker Flat (rkm 206) is entrenched in the Murray Gorge. Photo taken from the right bank looking east, main channel width approximately 170 m. (**b**) The lower Murray River exits the Murray Gorge at Wellington (rkm 78). Photo taken from the right bank looking east with the Wellington car ferry shown for scale, main channel width approximately 270 m. (**c**) The main body of Lake Alexandrina is so vast that the opposite shoreline cannot be seen by the naked eye. Photo taken looking east, distance to opposite shoreline approximately 37 km. (**d**) The final segment of the lower Murray River flows through the Goolwa channel (rkm 11) before reaching the Murray Mouth. Photo taken from the right bank looking east, approximate channel width 570 m. (**e**) Sir Richard Peninsula at Goolwa seen from a lookout point facing south east (rkm 12), where the lower Murray River meets the Southern Ocean. (**f**) Goolwa Beach on the Sir Richard Peninsula seen from the lookout point of (**e**) facing north west (rkm 12).

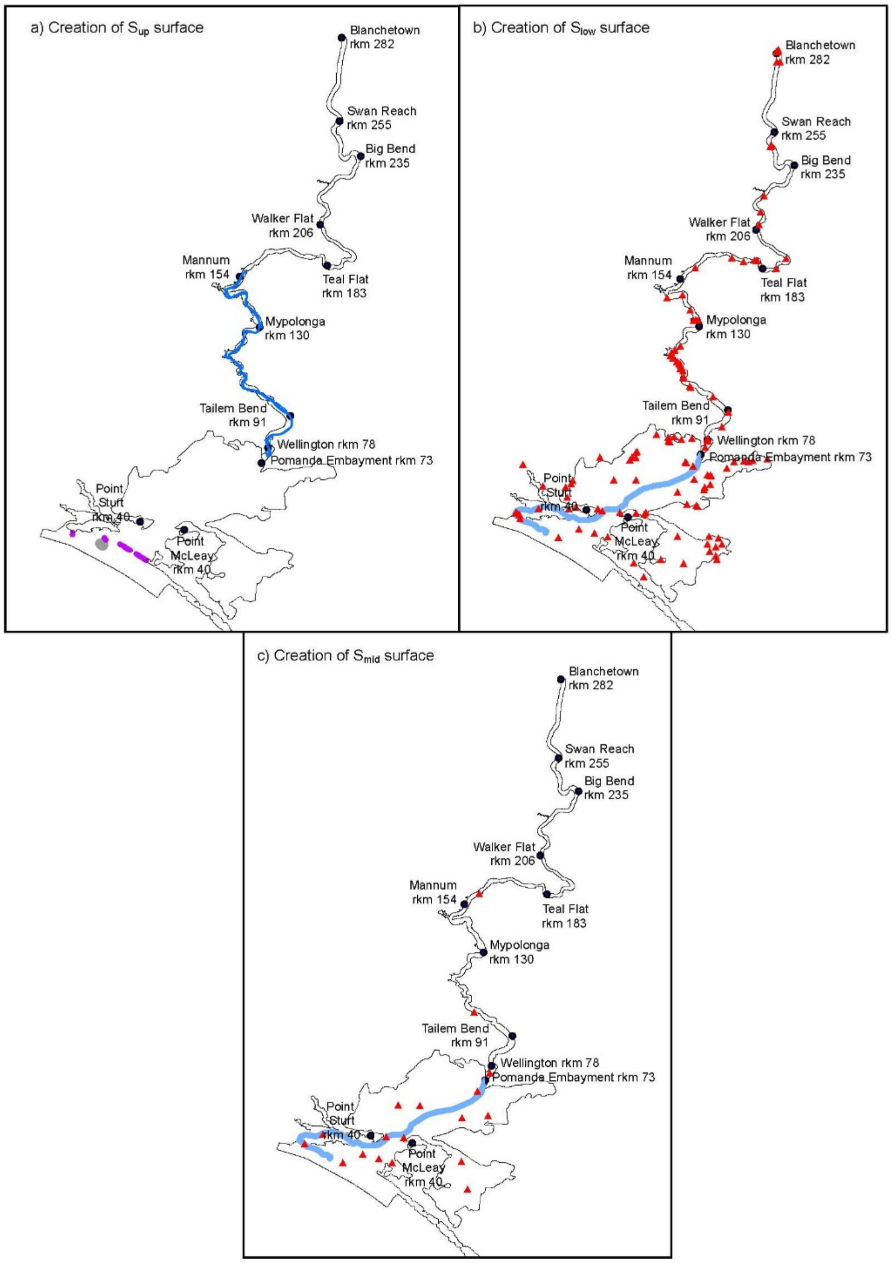

Figure S6: Overview of data used in the creation of three bathymetric surfaces. (**a**) Creation of the S_{up} surface involved manipulation of present day DEMs to remove man-made features including artificial levees (blue), Lock 1 at Blanchetown (rkm 282), Goolwa, Mundoo, Boundary Creek, Ewe Island and Tauwitchere barrages (purple), as well as Bird Island (grey), a modern flood tide deltaic island (*22*). (**b**) Creation of the S_{low} surface involved analysis of depth to the Coonambidgal-Monoman Formation transition, which is considered to mark the Pleistocene-Holocene boundary. Cores and CPTs analysed for creation of this surface (red) are given in Table S3. The location of the palaeo-Murray thalweg (blue) was inferred from data presented in Barnett (*9*). (**c**) Creation of the S_{mid} surface was resolved by subtracting regional sedimentation rates from the pre-regulation surface (*9, 11*) and dated sediment cores (red). Within the LMR and palaeo-Murray thalweg seaward (blue), a sedimentation rate of 0.69 mm/y was adopted (*9, 11*). All other elements were adjusted with a sedimentation rate of 0.16 mm/y (*9, 11*).

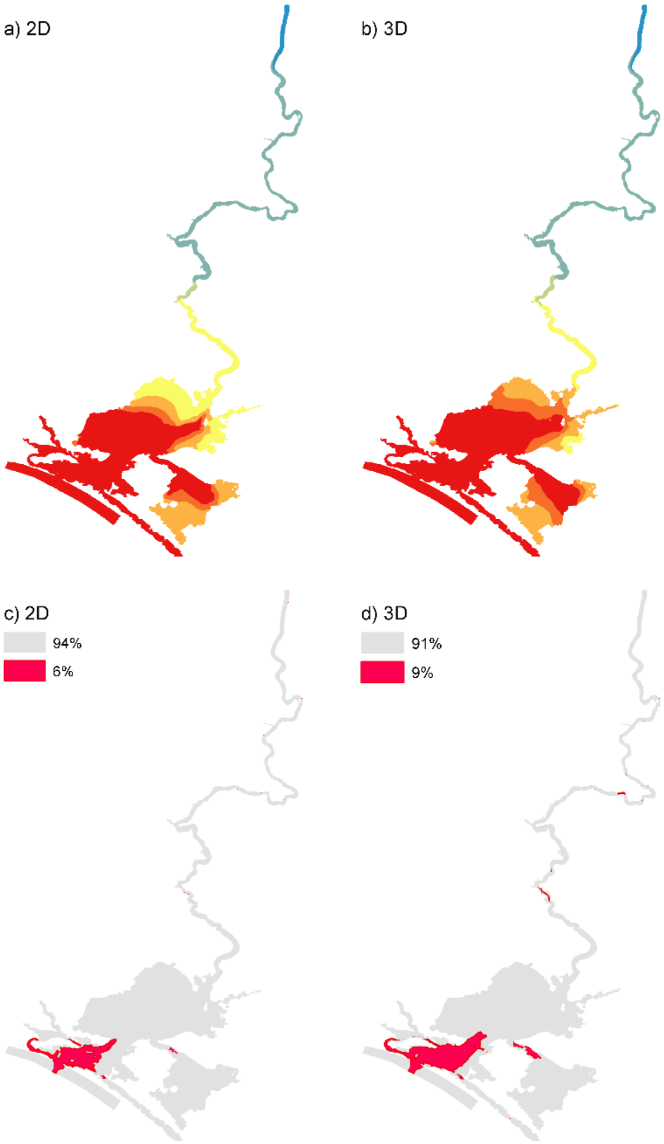

Figure S7: Comparison of $S_{mid}WL_2D_{av}B_{mod}$ 2D and 3D key outputs of maximum salinity and velocity magnitude. Maximum salinity reached in a best estimate Holocene highstand (**a**) 2D simulation is comparable to that of a (**b**) 3D simulation such that the classification of estuarine zonation remains consistent. 2D simulations provide a conservative approximation of 3D results. Salinity is measured based on the classification scheme of Tooley (*17*). Maximum velocity magnitude in a best estimate Holocene highstand (**c**) 2D simulation and a (**d**) 3D simulation. Areas are shaded red where maximum velocity > 0.3 m/s and therefore is not conducive to the deposition of a laminated silt-clay sequence (*19*, *20*, *21*). A 3% change in total area demonstrates the negligible difference in velocity magnitude between 2D and 3D simulations.

Table S1: The scenarios adopted in this study. Codes identify bathymetric surface (S_{up} = pre-modification condition, S_{mid} = highstand best estimate condition, S_{low} = Pleistocene-Holocene boundary condition), discharge (D_- = drought, D_{av} = pre-regulation average, D_+ = pre-regulation average with a flood event), and barrier morphology (B_0 = no barrier, B_+ and B_{++} = phases of chain-of-islands evolution (*3*, *4*, *12*, *13*), and B_{mod} = present day). A code and category has been assigned to each scenario to facilitate interpretation. Scenario categories are grouped based on sea-level and bathymetric surface.

Initial sea level at 2 m (with respect to 2014 sea level)					Initial sea level at 0 m (with respect to 2014 sea level)				
Bathymetric Surface	Discharge	Barrier Morphology	Scenario Code	Scenario Category	Bathymetric Surface	Discharge	Barrier Morphology	Scenario Code	Scenario Category
S_{up}	D_-	B_0	$S_{up}WL_2D_-B_0$	$\mathbf{S_{up}WL_2}$	S_{up}	D_-	B_0	$S_{up}WL_0D_-B_0$	$\mathbf{S_{up}WL_0}$
		B_+	$S_{up}WL_2D_-B_+$				B_+	$S_{up}WL_0D_-B_+$	
		B_{++}	$S_{up}WL_2D_-B_{++}$				B_{++}	$S_{up}WL_0D_-B_{++}$	
		B_{mod}	$S_{up}WL_2D_-B_{mod}$				B_{mod}	$S_{up}WL_0D_-B_{mod}$	
	D_{av}	B_0	$S_{up}WL_2D_{av}B_0$			D_{av}	B_0	$S_{up}WL_0D_{av}B_0$	
		B_+	$S_{up}WL_2D_{av}B_+$				B_+	$S_{up}WL_0D_{av}B_+$	
		B_{++}	$S_{up}WL_2D_{av}B_{++}$				B_{++}	$S_{up}WL_0D_{av}B_{++}$	
		B_{mod}	$S_{up}WL_2D_{av}B_{mod}$				B_{mod}	$S_{up}WL_0D_{av}B_{mod}$	
	D_+	B_0	$S_{up}WL_2D_+B_0$			D_+	B_0	$S_{up}WL_0D_+B_0$	
		B_+	$S_{up}WL_2D_+B_+$				B_+	$S_{up}WL_0D_+B_+$	
		B_{++}	$S_{up}WL_2D_+B_{++}$				B_{++}	$S_{up}WL_0D_+B_{++}$	
		B_{mod}	$S_{up}WL_2D_+B_{mod}$				B_{mod}	$S_{up}WL_0D_+B_{mod}$	
S_{mid}	D_-	B_0	$S_{mid}WL_2D_-B_0$	$\mathbf{S_{mid}WL_2}$	S_{mid}	D_-	B_0	$S_{mid}WL_0D_-B_0$	$\mathbf{S_{mid}WL_0}$
		B_+	$S_{mid}WL_2D_-B_+$				B_+	$S_{mid}WL_0D_-B_+$	
		B_{++}	$S_{mid}WL_2D_-B_{++}$				B_{++}	$S_{mid}WL_0D_-B_{++}$	
		B_{mod}	$S_{mid}WL_2D_-B_{mod}$				B_{mod}	$S_{mid}WL_0D_-B_{mod}$	
	D_{av}	B_0	$S_{mid}WL_2D_{av}B_0$			D_{av}	B_0	$S_{mid}WL_0D_{av}B_0$	
		B_+	$S_{mid}WL_2D_{av}B_+$				B_+	$S_{mid}WL_0D_{av}B_+$	
		B_{++}	$S_{mid}WL_2D_{av}B_{++}$				B_{++}	$S_{mid}WL_0D_{av}B_{++}$	
		B_{mod}	$S_{mid}WL_2D_{av}B_{mod}$				B_{mod}	$S_{mid}WL_0D_{av}B_{mod}$	
	D_+	B_0	$S_{mid}WL_2D_+B_0$			D_+	B_0	$S_{mid}WL_0D_+B_0$	
		B_+	$S_{mid}WL_2D_+B_+$				B_+	$S_{mid}WL_0D_+B_+$	
		B_{++}	$S_{mid}WL_2D_+B_{++}$				B_{++}	$S_{mid}WL_0D_+B_{++}$	
		B_{mod}	$S_{mid}WL_2D_+B_{mod}$				B_{mod}	$S_{mid}WL_0D_+B_{mod}$	
S_{low}	D_-	B_0	$S_{low}WL_2D_-B_0$	$\mathbf{S_{low}WL_2}$	S_{low}	D_-	B_0	$S_{low}WL_0D_-B_0$	$\mathbf{S_{low}WL_0}$
		B_+	$S_{low}WL_2D_-B_+$				B_+	$S_{low}WL_0D_-B_+$	
		B_{++}	$S_{low}WL_2D_-B_{++}$				B_{++}	$S_{low}WL_0D_-B_{++}$	
		B_{mod}	$S_{low}WL_2D_-B_{mod}$				B_{mod}	$S_{low}WL_0D_-B_{mod}$	
	D_{av}	B_0	$S_{low}WL_2D_{av}B_0$			D_{av}	B_0	$S_{low}WL_0D_{av}B_0$	
		B_+	$S_{low}WL_2D_{av}B_+$				B_+	$S_{low}WL_0D_{av}B_+$	
		B_{++}	$S_{low}WL_2D_{av}B_{++}$				B_{++}	$S_{low}WL_0D_{av}B_{++}$	
		B_{mod}	$S_{low}WL_2D_{av}B_{mod}$				B_{mod}	$S_{low}WL_0D_{av}B_{mod}$	
	D_+	B_0	$S_{low}WL_2D_+B_0$			D_+	B_0	$S_{low}WL_0D_+B_0$	
		B_+	$S_{low}WL_2D_+B_+$				B_+	$S_{low}WL_0D_+B_+$	
		B_{++}	$S_{low}WL_2D_+B_{++}$				B_{++}	$S_{low}WL_0D_+B_{++}$	
		B_{mod}	$S_{low}WL_2D_+B_{mod}$				B_{mod}	$S_{low}WL_0D_+B_{mod}$	

Table S2: Description of stratigraphic formations and soil combinations within the study area.

Formation Name	Description
Coonambidgal	Holocene alluvial clays and silts comprising the upper valley fill within the LMR
Monoman	Holocene to late-Pleistocene alluvial sands comprising the lower valley fill within the LMR
Saint Kilda	Holcene coastal marine sediment
Bridgewater	Middle-Pleistocene sands
Padthaway	Holocene to early-Pleistocene lacustrine sands, silts and clays
Molineaux Sand	Holocene to late-Pleistocene aeolian sands

Soil Combination Name	Description
Malcolm	Holocene estuarine, alluvial and lacustrine clays demarcating the greatest Holocene inundation extent of Lake Alexandrina

Table S3: Sedimentological data used to inform the creation of the S_{low} surface. Core logs (*9, 23, 24*) and cone penetrometer tests (*25*) were analysed for the transition from the Monoman to Coonambidgal Formation, which is interpreted to represent the approximate Pleistocene to Holocene boundary (S_{low}). Depths were adjusted relative to present day bathymetry and topography to give elevation in meters AHD.

rkm	X	Y	S_{low} Z (m AHD)	ID	Reference
3	305704	6064588	> 3.6	35	Barnett, 1993
10	300043	6067607	> 3.0	33	Barnett, 1993
18	305738	6069952	> 3.8	32	Barnett, 1993
25	317628	6080376	0.5	19	Barnett, 1993
30	315888	6069243	2.8	25	Barnett, 1993
30	315643	6066463	2.7	26	Barnett, 1993
32	317049	6064273	> 1.9	27	Barnett, 1993
35	321456	6063067	1.7	24	Barnett, 1993
36	325255	6062032	> 2.3	23	Barnett, 1993
39	323598	6069398	> 4.9	22	Barnett, 1993
40	321175	6077118	0.1	11	Barnett, 1993
40	320792	6081178	0.7	18	Barnett, 1993
40	321201	6075823	1.7	20	Barnett, 1993
43	328598	6068940	> 2.1	21	Barnett, 1993
46	335469.7	6068475	> -19.8	Narrung Ferry 3, #70435	SARIG
47	328808	6073753	> 4.8	15	Barnett, 1993
48	326907	6078155	1.3	16	Barnett, 1993
48	324273	6081247	0.4	17	Barnett, 1993
50	335386	6069807	> 0.8	14	Barnett, 1993
52	333115	6078087	1.5	13	Barnett, 1993
53	332968	6086037	1.1	9	Barnett, 1993
54	336659	6074639	> 4.8	12	Barnett, 1993
55	336077	6081656	1.4	10	Barnett, 1993
57	337929	6088161	> 1.3	8	Barnett, 1993
61	344830	6074599	> 2.0	6	Barnett, 1993
61	341063	6082300	0.2	7	Barnett, 1993
64	348312	6074474	> 0.3	5	Barnett, 1993
65	347387	6079188	-41.8	BH 2, #234134	SARIG
65	347477	6079083	> 2.0	3	Barnett, 1993
67	352233	6075279	> 1.5	4	Barnett, 1993
67	347256	6081111	> -20.6	BH 4, #234136	SARIG
69	349244	6082072	> 4.0	2	Barnett, 1993
76	352640	6087305	> 2.7	1	Barnett, 1993
78	353178	6089048	-14.8	Wellington	Hubble & De Carli, 2015
79	353311.8	6089252	-47.4	DEPT H & L G, #71577	SARIG
79	353169.8	6089073	-25.4	DEPT H & L G, #71668	SARIG
79	353346.8	6089207	-11.2	DEPT H & L G, #71581	SARIG
79	353112.8	6088951	-6.0	DEPT H & L G, #71669	SARIG
79	353306.8	6089250	> -35.0	DEPT H & L G, #71578	SARIG
79	353318.8	6089239	> -37.4	DEPT H & L G, #71580	SARIG
79	353318.8	6089239	> -30.7	DEPT H & L G, #71579	SARIG
79	353312	6089252	-48.5	6727-1105	Barnett, 1989
79	353170	6089073	> -22.4	6727-1196	Barnett, 1989
79	353233	6089803	> -2.7	Wellington East Marina	Hubble & De Carli, 2015
85	357350	6093006	> -0.4	Murray view Estates	Hubble & De Carli, 2015
85	357368	6093010	-21.7	Murray view Estates	Hubble & De Carli, 2015
88	358637.8	6093934	-0.6	Tailem Bend Pump 1, #71594	SARIG
92	359155.8	6097151	> -27.2	DEPT H & L G, #71665	SARIG

rkm	X	Y	S_{low} Z (m AHD)	ID	Reference
91.5	359156	6097151	> -27.4	6727-1193	Barnett, 1989
97	354771	6101444	-6.3	Westbrook	Hubble & De Carli, 2015
104	348576	6104196	-14.9	Riverglen Marina	Hubble & De Carli, 2015
104	348214	6104380	-27.2	Riverglen Marina	Hubble & De Carli, 2015
108	346634	6106805	-15.9	Bells Reserve Monteith	Hubble & De Carli, 2015
109	346395.8	6106955	-26.9	Monteith 1, #71337	SARIG
109	346396	6106955	-26.2	6727-865	Barnett, 1989
111	346364.8	6108814	-9.9	Swanport 6, #71336	SARIG
111.5	346365	6108814	-30.0	6727-864	Barnett, 1989
112	345553.8	6109505	-30.0	Swanport 4, #71334	SARIG
112	345554	6109505	> -29.2	6727-862	Barnett, 1989
113	345564	6110662	-3.9	Long Island Marina	Hubble & De Carli, 2015
113	345364	6110811	> -2.7	Long Island Marina	Hubble & De Carli, 2015
114	344665	6111528	-20.3	Long Island	Hubble & De Carli, 2015
114	344634	6111322	-12.7	Long Island Reserve	Hubble & De Carli, 2015
115	343532	6112394	> -1.9	Sturt Reserve, Murray Bridge	Hubble & De Carli, 2015
117	342847	6113083	-12.5	MB PUMP 1, #71347	SARIG
117	342824	6113068	-12.1	MB PUMP 2, #71348	SARIG
117	342807	6113023	-9.7	MB PUMP 3, #71349	SARIG
117	342732	6112903	-0.3	MD PUMP 7, #71354	SARIG
117	342847	6113083	> -20.2	6727-875	Barnett, 1989
117	342807	6113023	> -25.0	6727-877	Barnett, 1989
117	343129	6113921	-10.3	Thiele Reserve	Hubble & De Carli, 2015
119	344090.8	6114680	-21.9	CH2, #70979	SARIG
119	344067.8	6114650	-19.4	CH1, #70978	SARIG
119	344046.8	6114660	-9.7	PTH1, #70980	SARIG
119	344068	6114650	-18.9	6727-498	Barnett, 1989
119	344091	6114680	-21.3	6727-499	Barnett, 1989
119	344047	6114660	-9.4	6727-500	Barnett, 1989
120	345676	6115810	-13.1	Avoca Dell	Hubble & De Carli, 2015
133	350609	6123221	-9.6	6727-2201	Barnett, 1989
133	349935	6123145	-11.9	6727-2205	Barnett, 1989
133	349305	6123011	-19.5	6727-2214	Barnett, 1989
136	348350	6125995	-6.9	Woodlane Reserve	Hubble & De Carli, 2015
142	346193	6130131	-14.2	Wall Flat	Hubble & De Carli, 2015
147	341939	6129444	> -0.3	Neeta Irrigation Area	Hubble & De Carli, 2015
159	349749	6137775	-15.8	East Front Rd	Hubble & De Carli, 2015
160	349204.8	6137915	-11.8	LOWER MURRAY DAM 5 1, #73409	SARIG
161	350989.8	6138882	-4.0	LOWER MURRAY DAM 4 1, #73297	SARIG
172	360167	6140549	-9.2	Younghusband	Hubble & De Carli, 2015
175	363437	6139565	-11.9	Younghusband	Hubble & De Carli, 2015
179	366522	6139908	-10.3	6828-427	Barnett, 1989
180	367076.9	6139963	-12.9	TEAL FLAT PD11, #85363	SARIG
180	367141.8	6139778	-13.5	TEAL FLAT PD28, #85308	SARIG
180	367117	6139848	> -7.8	6828-431	Barnett, 1989
180	367227	6139833	-6.0	6828-434	Barnett, 1989
187	372520	6137668	-7.4	BowHill	Hubble & De Carli, 2015
194	375342	6140438	-15.6	Purnong	Hubble & De Carli, 2015

rkm	X	Y	S_{low} Z (m AHD)	ID	Reference
208	367778	6149959	-4.4	Scrubby Flat	Hubble & De Carli, 2015
213	368198	6153555	-10.5	Walkers Flat	Hubble & De Carli, 2015
221	369210	6158135	-9.8	Wongulla	Hubble & De Carli, 2015
250	371056	6172095	1.1	6828-578	Barnett, 1989
250	370985	6172001	-11.7	6828-579	Barnett, 1989
250	371143	6172150	-7.9	6828-580	Barnett, 1989
250	371381	6172318	-5.6	6828-581	Barnett, 1989
250	371232	6172245	-8.2	6828-582	Barnett, 1989
251	370984.9	6172001	-15.8	Swan Reach 2, #85459	SARIG
251	371142.9	6172150	-11.0	Swan Reach 3, #85458	SARIG
251	370896.9	6171774	-10.4	PH5, #85384	SARIG
251	371055.9	6172095	-8.1	Swan Reach 1, #85458	SARIG
251	371497.9	6172390	-6.1	Swan Reach 10, #85463	SARIG
251	371380.9	6172318	-5.8	Swan Reach 7, #85461	SARIG
251	370896.9	6171774	-3.5	PH6, #85385	SARIG
280	373624	6195800	-3.1	6829-833	Barnett, 1989
280	372657	6195846	7.3	6829-829	Barnett, 1989
284	372922	6198708	-2.5	Blanchetown Bridge 5, #85879	SARIG
284	372612	6198638	0.0	Blanchetown Bridge 12, #85835	SARIG
284	372637	6198648	0.1	Blanchetown Bridge 13, #85836	SARIG
284	372577.1	6198633	0.3	Blanchetown Bridge 1, #85833	SARIG
284	372882	6198698	1.1	Blanchetown Bridge 10, #85878	SARIG
284	372577	6198628	1.2	Blanchetown Bridge 1A, #85834	SARIG
284	372677.1	6198653	2.2	Blanchetown Bridge 2, #85837	SARIG
284	373142	6198758	3.2	Blanchetown Bridge 11, #85881	SARIG
284	372982	6198723	5.1	Blanchetown Bridge 6, #85880	SARIG
284	372562	6198628	9.2	Blanchetown Bridge 9, #85832	SARIG
284	372547	6198623	18.9	Blanchetown Bridge 7, #85831	SARIG
283.5	372577	6198628	3.5	6829-170	Barnett, 1989
283.5	372677	6198653	2.8	6829-173	Barnett, 1989
283.5	372772	6198673	0.2	6829-212	Barnett, 1989
283.5	372922	6198708	6.7	6829-215	Barnett, 1989
284	373148	6199263	> -3.9	6829-1380	Barnett, 1989

References

1. J. French, A. Payo, B. Murray, J. Orford, M. Eliot, P. Cowell, Appropriate complexity for the prediction of coastal and estuarine geomorphic behaviour at decadal to centennial scales. *Geomorphology* **256**, 3-16 (2016).
2. H.J. de Vriend, M. Capobianco, T. Chesher, H.E. de Swart, B. Latteux, M.J.F. Stive, Approaches to long-term modelling of coastal morphology: A review. *Coastal Engineering* **21** (1), 225-269 (1993).
3. R. A. Luebbers, The Coorong Report: An archaeological survey of the Northern Coorong, Prepared for the South Australian Department for Environment and Planning, (1982).
4. C. J. de Mooy, Notes on the geomorphic history of the area surrounding Lakes Alexandrina and Albert. *Trans. Roy. Soc. S. Aust.* **82**, 99-118 (1959).
5. J. H. Cann, R. P. Bourman, E. J. Barnett, Holocene Foraminifera as Indicators of Relative Estuarine-Lagoonal and Oceanic Influences in Estuarine Sediments of the River Murray, South Australia. *Quat. Res.* **53**, 378-391 (2000).
6. R. W. Dalrymple, B. A. Zaitlin, R. Boyd, Estuarine facies models; conceptual basis and stratigraphic implications. *J. Sediment. Res.* **62**, 1130-1146 (1992).
7. A. Ladson, S. Lang, B. Anderson, I. D. Rutherfurd, An Australian Handbook of Stream Roughness Coefficients, paper presented at the 28th Hydrology and Water Resources Symposium, Wollongong, 10-14 November (2003).
8. R. Hudson, Modelling Investigation into the Wellington 'Virtual Weir' Concept: Project summary report, BMT WBM, Prepared for the Murray Darling Basin Authority (MDBA) (MDBA, 2010).
9. E. J. Barnett, Recent Sedimentary History of Lake Alexandrina and the Murray Estuary. The Flinders University of South Australia (1993).
10. C. Von der Borch, M. Altmann, Holocene stratigraphy and evolution of the Cooke Plains Embayment, a former extension of Lake Alexandrina, South Australia. *Trans. Roy. Soc. S. Aust.* **103**, 69-78 (1979).
11. J. Fluin, A diatom-based palaeolimnological investigation of the lower Murray River (south east Australia), School of Geography and Environmental Science, Monash University (2002).
12. N. Harvey, Holocene Coastal Evolution: Barriers, Beach Ridges, and Tidal Flats of South Australia. *J. Coast. Res.*, 90-99 (2006).
13. B. Bourman, C. V. Murray-Wallace, Holocene evolution of a sand spit at the mouth of a large river system: Sir Richard Peninsular and the Murray Mouth, South Australia. *Z. Geomorphol. Suppl.* **81**, 63-83 (1991).
14. R. P. Bourman, C. V. Murray-Wallace, A. P. Belperio, N. Harvey, Rapid coastal geomorphic change in the River Murray Estuary of Australia. *Mar. Geol.* **170**, 141-168 (2000).
15. N. Harvey, R. Bourman, J. Kris, Evolution of the Younghusband Peninsula, South Australia: New evidence from the northern tip. *South Australian Geographical Journal* **105**, 37-50 (2006).
16. M. Disspain, L.A. Wallis, B.M. Gillanders, Developing baseline data to understand environmental change: a geochemical study of archaeological otoliths from the Coorong, South Australia. *J Archaeol Sci* **38**, 1842-1857 (2011).
17. M. J. Tooley, Sea-level Changes: Northwest England during the Flandrian stage. (Clarendon Press, 1978).
18. B. A. Zaitlin, R. W. Dalrymple, R. Boyd, The stratigraphic organization of incised-valley systems associated with relative sea-level change, [R. W. Dalrymple, R. Boyd, B. A. Zaitlin, (eds.)] Incised-Valley Systems: Origin and Sedimentary Sequences, Special Publication No. 51, pp. 45-60 (SEPM (Society for Sedimentary Geology), Tulsa, Oklahoma, U.S.A., 1994).
19. J. Schieber, Z. Yawar, A new twist on mud deposition - mud ripples in experiment and rock record. *The Sedimentary Record* **7**, 4-8 (2009).
20. J. Schieber, J. Southard, K. Thaisen, Accretion of Mudstone Beds from Migrating Floccule Ripples. *Science* **318**, 1760-1763 (2007).

21. J. H. Baas, J. L. Best, J. Peakall, Predicting bedforms and primary current stratification in cohesive mixtures of mud and sand. *J. Geol. Soc.* **173**, 12-45 (2016).
22. K.F. James, B. Bourman, N. Harvey, Rapid evolution of a flood tide deltaic island in the River Murray estuary, South Australia: A canary in the cage of river management. *J Coast Res* **31**, 5, 1103-1119 (2015).
23. S.R. Barnett, The hydrogeology of the Murray Basin in South Australia with special reference to the alluvium of the River Murray floodplain, School of Earth Sciences, Flinders University of South Australia (1989).
24. South Australian Resources Information Gateway (SARIG). Department of State Development, the Government of South Australia. Drillholes: All mineral drillholes. https://map.sarig.sa.gov.au/
25. T. C. T. Hubble, E. De Carli, Mechanisms and Processes of the Millennium Drought River Bank Failures: Lower Murray River, South Australia, Goyder Institute for Water Research Technical Report (Adelaide, South Australia, 2015).
